# Regulating Zinc Anode Interface with an Environmental Biomass‐Derived Additive for Long‐Lifespan Aqueous Batteries

**DOI:** 10.1002/advs.202522511

**Published:** 2026-01-07

**Authors:** Bingbo Ni, Qian Wang, Qiusheng Ma, Junhui Cheng, Xingxing Gu, Guangyin Li

**Affiliations:** ^1^ Northeast Institute of Geography and Agroecology Chinese Academy of Sciences Changchun China; ^2^ Chongqing Key Laboratory of Environmental Catalysis College of Environment and Resources Chongqing Technology and Business University Chongqing China; ^3^ University of Chinese Academy of Sciences Beijing China; ^4^ College of Hydrology and Water Resources Hohai University Nanjing China; ^5^ School of Geographic and Oceanographic Science Nanjing University Nanjing China

**Keywords:** additive, environmental biomass, long‐lifespan, zinc anode

## Abstract

Aqueous zinc‐based batteries face critical stability issues at the zinc metal anode, primarily manifested as uncontrolled dendrite growth, hydrogen evolution reaction, and corrosion. To address these issues in an eco‐friendly manner, we report a biomass‐derived additive, 3‐acetylamino‐5‐acetylfuran (3A5AF), synthesized from chitin, which features abundant polar N/O functional groups. Even at an ultralow concentration (0.3 mg mL^−^^1^), 3A5AF could restructure the solvation shell of Zn^2^^+^ and establish a protective layer on the anode surface, thereby curbing undesirable side reactions and guiding the uniform deposition of zinc. This stabilization strategy endows the Zn||Zn symmetric cell with robust longevity, achieving a cycle life exceeding 2700 h under 1 mA cm^−^^2^ and 1 mAh cm^−^^2^. Even when subjected to a demanding current density of 4 mA cm^−^^2^, the cell maintains stable operation for 2400 h. The practical utility was further confirmed in Zn||I_2_ full cells, which delivered a reversible capacity of 192.6 mAh g^−1^ following 1000 cycles at 0.5 A g^−^^1^ and, at 8 A g^−^^1^, sustained 20 000 cycles with merely a 6.1% capacity loss (93.9% retention). This work highlights the promise of sustainable biomass‐derived additives in developing high‐performance and green aqueous zinc batteries.

## Graphical abstract

A biomass‐derived additive synthesized from chitin, 3‐acetylamino‐5‐acetylfuran (3A5AF), resolves key stability issues such as uncontrolled dendrite growth, hydrogen evolution reaction, and corrosion of zinc anodes by reconstructing the solvation structure of the electrolyte and protecting the anode interface.

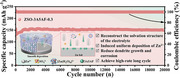

## 1 Introduction

The increasingly severe global climate change challenges and environmental pollution problems urgently require humans to expedite the transition to cleaner, lower‐carbon, safer and more efficient energy infrastructure for sustainable development [1, 2]. Sustainable development increasingly relies on the large‐scale harnessing of renewable energy sources, particularly wind and solar power [3]. However, the intermittent and fluctuating characteristics of renewable energy pose urgent demands for efficient, reliable, and economically viable large‐scale energy storage technologies [4]. Currently, the predominance of lithium‐ion batteries (LIBs) in markets ranging from portable electronics to electric vehicles is underpinned by their compelling attributes, including high energy density, reliable cycle stability, and superior energy efficiency [5, 6]. They are also actively expanding into the grid‐level energy storage market. However, they face significant challenges in more widespread applications: the global uneven distribution of lithium resources and the rising raw material costs limit their economic and scalability; more critically, the inherent flammability of the organic electrolyte system brings serious thermal runaway safety risks, threatening the safe operation of large‐scale energy storage systems [7]. Therefore, it is crucial to explore new energy storage systems that are environmentally friendly, inherently safe, resource‐rich, and cost‐effective.

Despite attractive features such as high theoretical capacity (820 mAh g^−^^1^), low potential (−0.76 V vs. SHE), abundant zinc resources, and the inherent safety of aqueous electrolytes, aqueous zinc‐iodine (Zn‐I_2_) batteries face significant challenges rooted in electrode instability. The cathode chemistry, based on the I_2_/I^−^ redox couple, is plagued by polyiodide dissolution and shuttling (e.g., I_3_^−^), causing active material loss, self‐discharge, and low Coulombic efficiency [8]. Meanwhile, the zinc anode is hindered by dendrite growth, hydrogen evolution, and corrosion during cycling [9]. These anode issues can lead to internal short circuits, depletion of electrolyte and active material, and gas evolution [10]. Crucially, these anode‐related interface failures not only limit zinc cycling performance but can also exacerbate the cathode's shuttle effect via non‐uniform electric fields and side reactions, creating an interrelated degradation cycle [11].

To address the aforementioned key challenges, researchers have explored solutions from the perspectives of the positive and negative electrodes respectively. In the positive electrode aspect, the core strategy focuses on inhibiting the dissolution and shuttle of polyiodides, mainly through designing high‐performance host materials (such as porous carbon, metal‐organic frameworks, conductive polymers, etc.) for physical confinement [12], or introducing sites with strong chemical adsorption/catalytic functions (such as heteroatom doping, polar groups, metal compounds) to anchor polyiodides and enhance reaction kinetics [13, 14]. In the negative electrode/interface aspect, efforts are directed toward solving issues such as zinc dendrites, hydrogen evolution reaction (HER), and corrosion, mainly through strategies such as constructing artificial interface protective layers (such as inorganic coatings, organic polymer membranes) [15, 16], designing 3D conductive host structures to guide uniform zinc deposition [17], and optimizing the properties of separators and electrolytes [18, 19]. Among the various strategies for optimizing the negative electrode interface, electrolyte engineering, especially the introduction of functional additives, is considered to be a highly promising approach owing to its simplicity of operation, low cost, and the ability to effectively guide uniform zinc deposition, inhibit dendrite growth, reduce hydrogen evolution and corrosion, by mechanisms such as adjusting the solvation structure of Zn^2^^+^, forming protective adsorption layers on the zinc surface, or suppressing side reactions [11, 20].

Meanwhile, with the increasing emphasis on the concept of sustainable chemistry, the design of electrolyte additives is actively moving toward environmentally friendly and green renewable directions [20]. In this context, bio‐derived additives have emerged as an important source for developing new and sustainable electrolyte additives due to their significant environmental friendliness (low toxicity, biodegradability), abundant and renewable raw material sources, and strong molecular structure tunability (facilitating molecular design for zinc anode protection requirements) [21, 22]. For instance, in the work of Quan et al. [23], food‐grade sorbitol (C_6_H_14_O_6_) was introduced as an electrolyte additive. Sorbitol contains abundant hydroxyl groups and can strongly interact with water molecules and zinc electrodes, thereby restructuring the primary solvation sheath of Zn^2^^+^ ions and broadening the working potential of the electrolyte. Liu et al. introduced a natural binary additive composed of saponins and fenchaldehyde [24] to enhance the corrosion resistance of the zinc anode in zinc sulfate (ZnSO_4_) aqueous solution and the stability of the interface SEI layer. The results showed that the improvement in zinc anode interface stability was mainly due to the abundant hydroxyl and carboxyl groups of saponins serving as “anchors”, and the saponin additive reshaped the EDL structure, enhancing the chemical adsorption of the fenchaldehyde molecule within the EDL, enabling the symmetric battery to stably cycle for 3400 h at 1 mA cm^−2^.

**FIGURE 1:**
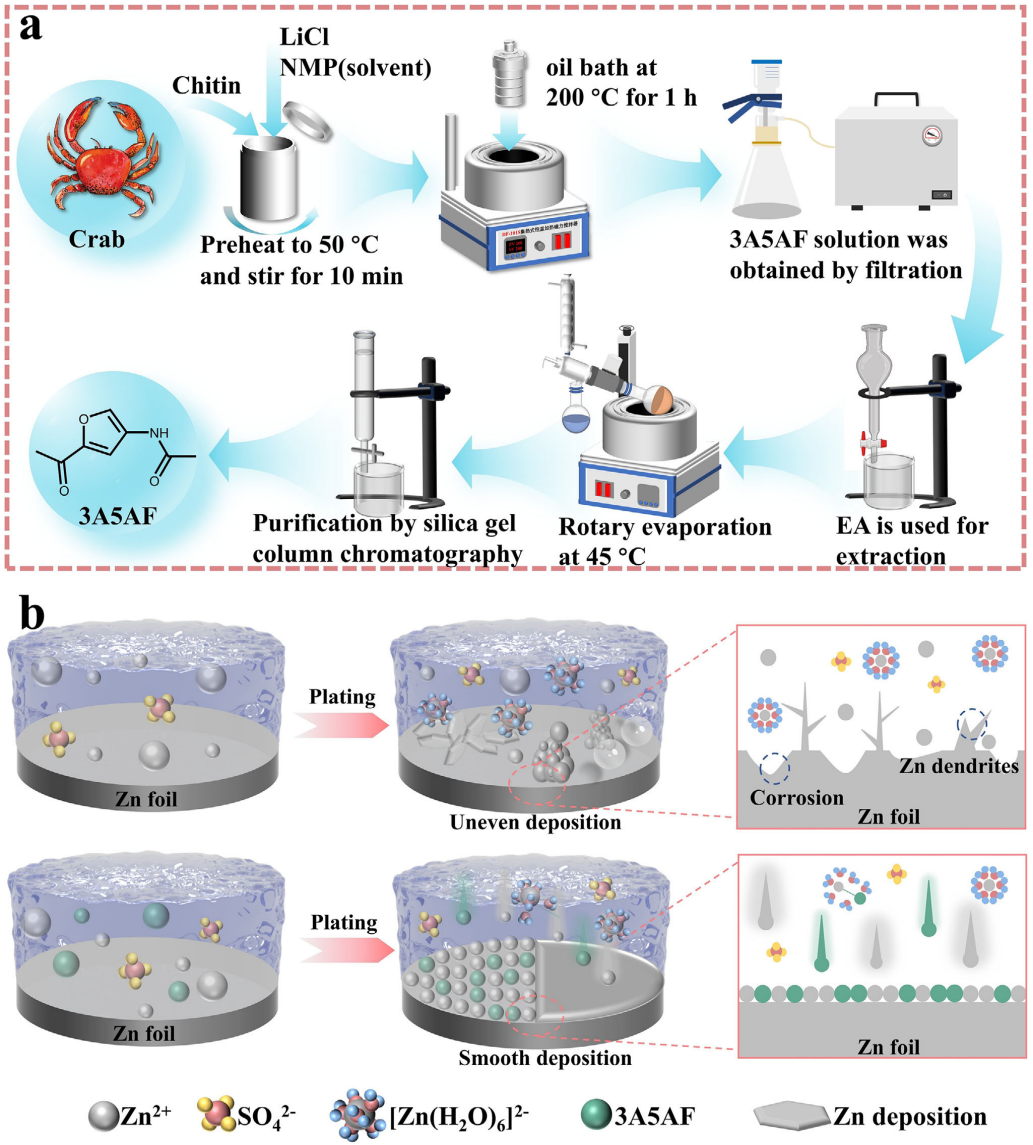
(a) Preparation flowchart of 3A5AF. (b) Mechanism diagram of the Zn deposition process in blank ZSO and ZSO + 3A5AF electrolytes.

Biomass additive provides a new idea with both high performance and ecological sustainability to address the core challenge of interface stability of zinc anodes. Inspired by this, a biomass additive, 3‐acetylamino‐5‐acetylpyran (3A5AF), derived from chitin (Figure 1a), which is rich in polar nitrogen and oxygen functional groups, afforded effective protection to the Zn anode. As illustrated in Figure 1b, in the blank ZSO electrolyte, the Zn anode is highly unstable and prone to corrosion and passivation, as well as the growth of dendrites. In contrast, with adding 3A5AF in ZSO electrolyte, the zinc anode exhibits remarkably boosted stability, which is because the rich polar functional groups in 3A5AF can effectively reorganize the solvation sphere of Zn^2^^+^ ions through hydrogen bonding interactions, reducing the activity of H_2_O molecules, thereby significantly reducing the occurrence of uncontrollable HER side reactions. Moreover, the abundant lone pairs of electrons in 3A5AF give it stronger zinc‐affinity, enabling 3A5AF molecules to strongly anchor onto the Zn anode surface and offer numerous sites for Zn^2^^+^ nucleation, thereby inducing uniform deposition of Zn^2+^. Thanks to these advantages, the Zn||Zn symmetric battery with only 0.3 mg mL^−1^ 3A5AF additive exhibits a lifespan of over 2700 h (1 mA cm^−2^ and 1 mAh cm^−2^), and its cycle life could still reach 2400 h even at a high current density of 4 mA cm^−2^. In the Zn||Cu half‐cell, a high reversible electroplating/deplating performance of an average CE of 99.5% is achieved in over 800 cycles. Additionally, the 3A5AF‐based Zn||I_2_ full cell demonstrated excellent capacity retention of 93.0% over 1000 cycles at 0.5 A g^−^^1^. Notably, at an ultra‐high rate of 8 A g^−^^1^, it sustained a capacity of 149.6 mAh g^−^^1^ through 20 000 cycles, showcasing exceptional durability. What is more remarkable is that the Zn‐I_2_ pouch battery could also achieve stable cycling for 200 times at a current density of 0.2 A g^−1^.

## 2 Results and Discussions

**FIGURE 2:**
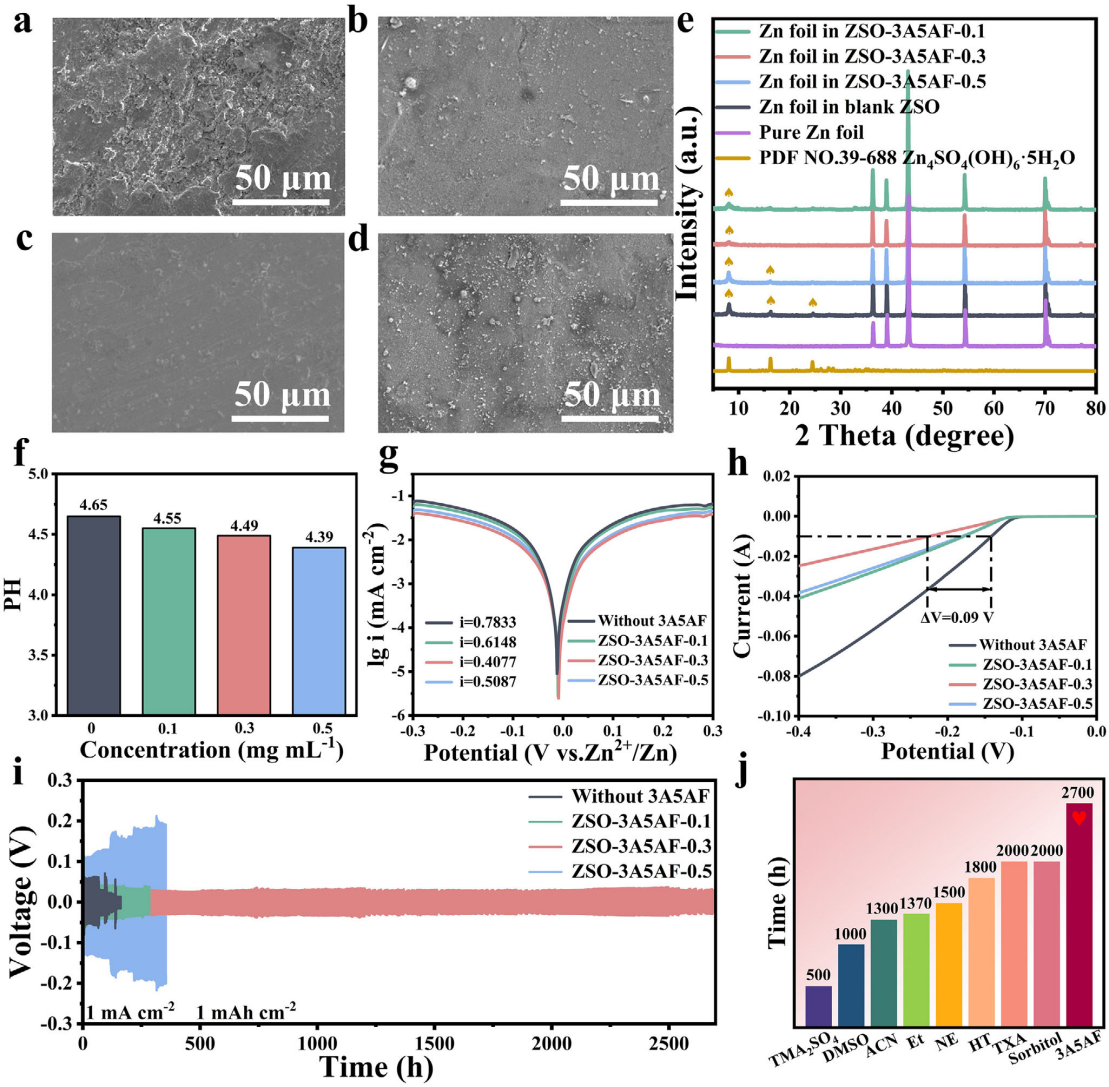
Verification and Concentration Optimization of 3A5AF Additive for Stabilizing Zinc Anode. SEM images of the zinc foil surface after being immersed in (a) ZSO, (b)ZSO‐3A5AF‐0.1, (c) ZSO‐3A5AF‐0.3, and (d) ZSO‐3A5AF‐0.5 electrolyte solutions. (e) XRD spectra of the zinc foil after being immersed in ZSO and various 3A5AF additive‐based solutions. (f) The pH values of the electrolytes with different concentrations of 3A5AF. (g) Tafel curves of Zn||Zn symmetric cells under different concentrations of 3A5AF‐based electrolyte. (h) The LSV curves of Zn||Ti asymmetric batteries under different 3A5AF concentrations of electrolyte. (i) The cycling performance of the Zn||Zn symmetric battery in different electrolytes with different concentrations of 3A5AF. (j) The comparison of the cycling performance of Zn||Zn symmetric battery at the ofptimal 3A5AF concentration with previous studies.

To verify the protective effect of 3A5AF on zinc anodes, four zinc anodes of the same size were respectively immersed in ZSO electrolyte with different contents of 3A5AF for 12 h. The morphological changes are shown in Figure 2. Upon exposure to the additive‐free ZSO electrolyte, the zinc anode showed pronounced unevenness and corrosion damage, and there are a large number of irregular large pieces of by‐products (Figure 2a). In contrast, the surface of the zinc negative electrode after being immersed in the ZSO‐3A5AF electrolyte is relatively compact and smooth (Figure 2b–d), with less corrosion and significantly fewer by‐products. Especially when using the 3A5AF electrolyte containing 0.3 mg mL^−1^, the surface is the smoothest and the by‐products show the least amount (Figure 2c). This morphological variation is due to the inadequate surface coverage provided by the low concentration of 3A5AF molecules, resulting in local areas still undergoing side reactions [25]. On the other hand, elevated concentrations of 3A5AF induce a drop in the pH value of the electrolyte, as evidenced in Figure 2f and Figure S1, causing an increase in the number of free H^+^, which in turn promotes the corrosion of the zinc anode [10]. The corresponding X‐ray diffraction (XRD) results also confirmed this conclusion. As shown in Figure 2e, the XRD spectra of the Zn foil after soaking in the blank ZSO solution showed strong diffraction peaks at 8°, 16° and 24°, confirming the formation of the corresponding by‐product Zn_4_SO_4_(OH)_6_·5H_2_O. The appearance of these diffraction peaks corroborates that severe side reactions took place on the zinc anode in the pure ZSO electrolyte. In contrast, these characteristic peaks were significantly attenuated in the ZSO‐3A5AF electrolyte. And when the content of 3A5AF was 0.3 mg mL^−1^, almost no diffraction peaks belonging to the by‐product Zn_4_SO_4_(OH)_6_·5H_2_O were observed. This indicates that the appropriate 3A5AF electrolyte additive has a positive impact on the corrosion resistance of the Zn negative electrode, effectively inhibiting the formation of the by‐product [20].

Subsequently, the anti‐corrosion effect of 3A5AF was further studied via Tafel and LSV analyses. As plotted in Figure 2g, a significant drop in corrosion current density from 0.78 to 0.41 mA cm^−^^2^ was observed with the initial increase of 3A5AF, followed by a subsequent rise to 0.51 mA cm^−^^2^ at excessive concentrations. When a small amount of 3A5AF was added, the decrease in corrosion current could be attributed to the effective inhibition of the Zn negative electrode corrosion by the 3A5AF additive [10]. However, as more 3A5AF was added, it was necessary to consider that 3A5AF itself is acidic, which would reduce the pH value of the electrolyte and increase the concentration of free H^+^. This would promote the occurrence of hydrogen evolution corrosion. Therefore, the corrosion current density would increase again [26]. This explanation is further supported by the HER measurements shown in Figure 2h, which exhibit a consistent trend. As the concentration of the additive increased, the onset potential of HER first increased and then decreased. At 0.3 mg mL^−1^, hydrogen evolution corrosion was most effectively inhibited.

To verify this concentration‐dependent trend identified by the corrosion tests, the cycling stability of Zn||Zn symmetric cells with electrolytes of varying 3A5AF concentrations was evaluated. At current densities of 1 mA cm^−^^2^ and 1 mAh cm^−^^2^, the Zn symmetrical battery based on blank ZSO electrolyte formed dendrites due to uneven deposition of Zn^2^^+^ [27], which easily pierced the separator and caused an internal short circuit, resulting in failure approximately 150 h later, accompanied by severe voltage fluctuations (Figure 2i). After the addition of 3A5AF, the cycling performance of the Zn||Zn symmetric cell was significantly improved. Especially when the addition amount of 3A5AF was 0.3 mg mL^−1^, the battery achieved the optimal cycling life, demonstrating a long‐term stability of over 2700 h. However, when the concentration of 3A5AF continued to increase, the voltage‐time curve showed that the total voltage lag phenomenon of the zinc anode became more obvious, and the voltage polarization intensified, which might be caused by side reactions and dendrite growth [28]. Therefore, 0.3 mg mL^−1^ was determined as the optimal concentration of this additive.

The superiority of the optimal ZSO‐3A5AF‐0.3 electrolyte was further confirmed under high current densities and deposition capacities. The Zn||Zn symmetric cell not only demonstrated a stable lifespan of 2000 h at 2 mA cm^−^^2^ and 2 mAh cm^−^^2^ (Figure S2) but also achieved remarkable stability over 2400 h at 4 mA cm^−^^2^. This performance starkly contrasts with the rapid failure (∼160 h) of the blank ZSO cell under the same harsh conditions, underscoring the effectiveness of 3A5AF in suppressing dendrites. These results indicate that 3A5AF significantly promotes the cycling performance of the zinc electrode. It is worth noting that the cycling performance based on the ZSO‐3A5AF‐0.3 electrolyte is superior to many other electrolyte additives reported recently (Figure 2j; Table S1). Following the assessment of cycling stability, the CE of Zn||Cu half‐cells was evaluated to provide complementary insight into the reversibility of zinc deposition at the optimal 3A5AF concentration. As illustrated in Figure S3a, the average CE of the ZSO‐based Zn||Cu battery is only 89.65%, and it can only cycle less than 100 cycles. However, the average CE of the ZSO‐3A5AF‐0.3 electrolyte‐based Zn||Cu battery was as high as 99.51%, and it can cycle more than 800 cycles. Meanwhile, it can be observed from the voltage capacity curves that the Zn||Cu half‐cell using ZSO electrolyte exhibited significant fluctuations during the cycling process (Figure S3b), while the ZSO‐3A5AF‐0.3 electrolyte‐based Zn||Cu battery remained stable during the cycling process and demonstrated significant reduction in polarization (Figure S3c) [29]. All these results indicate the high reversibility of the zinc deposition/stripping process by utilizing the 3A5AF additive.

**FIGURE 3:**
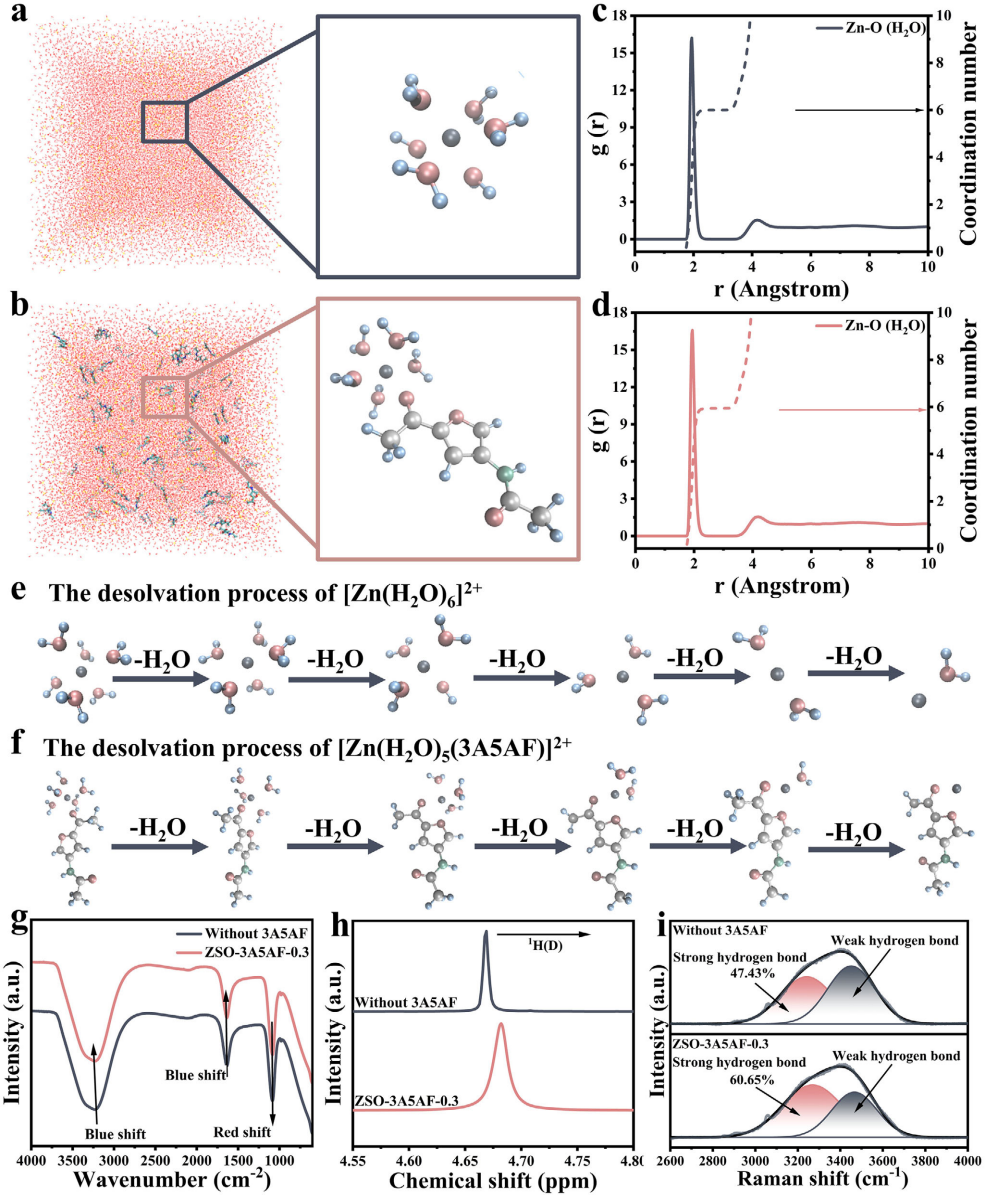
Theoretical and experimental verification of 3A5AF's influence on the solvation structure of the electrolyte solution. Three‐dimensional snapshots of the MD simulation in (a) ZSO electrolyte and (b) ZSO‐3A5AF‐0.3 electrolyte. (c) RDFs obtained from the MD simulation in the ZSO electrolyte. (d) RDFs obtained from the MD simulation in the ZSO‐3A5AF‐0.3 electrolyte. (e) The desolvation molecular geometries of [Zn(H_2_O)_6−x_]^2+^ in ZSO and (f) The desolvation molecular geometries of [Zn(H_2_O)_5−x_(3A5AF)]^2+^ in ZSO‐3A5AF‐0.3 electrolyte, respectively, x ranging from 1 to 5. Blank ZSO and ZSO‐3A5AF‐0.3 electrolytes' (g) ATR‐FTIR spectra, (h) ^1^H NMR spectra, and (i) Raman spectra.

Given the remarkable improvement in cycling stability, it is essential to understand the fundamental interactions at play. First, the primary solvation sheath (PSS) structures in ZSO‐3A5AF and blank ZSO electrolytes were analyzed through molecular dynamics (MD) simulations. The computational results indicated that after complete stabilization in a pure ZSO environment, the PSS of Zn^2+^ contained six H_2_O molecules (Figure 3a). Upon introducing 3A5AF into the electrolyte, one H_2_O molecule in the PSS was replaced by one 3A5AF molecule (Figure 3b). The continuous results of MD (Figure S4) show that the configuration of 3A5AF in the Zn^2^^+^ solvated shell does not change significantly during the simulation time, which provides direct evidence for the stable existence of 3A5AF in the primary solvated sheath of Zn^2^^+^ [30]. Meanwhile, based on the MD simulation results, the corresponding radial distribution functions and coordination numbers were obtained. In the ZSO‐3A5AF electrolyte system (Figure 3d), the Zn─O peak is observed at approximately 1.94 Å, consistent with that in the blank ZSO electrolyte system (Figure 3c). This peak can be attributed to water molecules (H_2_O) present in the PSS. Notably, the average coordination number of Zn─O (H_2_O) in the PSS decreased from 6.0 in the pure ZSO electrolyte to 5.94 in the ZSO‐3A5AF electrolyte. This reduction suggests that the incorporation of 3A5AF molecules disrupts the original solvation sheath structure. Therefore, it can be concluded that 3A5AF effectively modulates the number of coordinated water molecules in the solvation environment [31]. Moreover, based on the MD results, the desolvation process of the hydrated [Zn(H_2_O)_5‐x_(3A5AF)]^2+^ and [Zn(H_2_O)_6‐_ *_x_*]^2+^ (*x* = 1–5) ions is further presented in Figure 3e,f, complemented by a quantitative presentation of the desolvation energies in Figure S5. The stepwise desolvation energy barriers for [Zn(H_2_O)_5‐x_(3A5AF)]^2^^+^ were markedly reduced relative to those of the pure aqueous solvation structure [Zn(H_2_O)_6‐x_]^2^^+^, proving that 3A5AF can effectively coordinate with the central Zn^2+^ ion and make the desolvation process easier. Meanwhile, a lower desolvation energy barrier means that Zn^2+^ ions are more likely to transform from a solvated state to depositable metallic zinc at the electrode interface, thereby enhancing the rate and reversibility of the zinc deposition reaction, and thus improving the rate performance and long cycle life [32].

Further infrared, Raman, and nuclear magnetic resonance analyses were employed to confirm that the electrolyte underwent a change in its solvation structure after the introduction of 3A5AF. As shown in the infrared spectrum in Figure 3g, a slight redshift occurs in the SO_4_^2−^ vibration at approximately 1100 cm^−1^, indicating a weakening of the interaction between Zn^2+^ and SO_4_^2−^. At the same time, the characteristic peaks near 3200 cm^−^^1^ (O─H stretch) and 1600 cm^−^^1^ (O─H bend) showed a concomitant blue shift as the concentration of 3A5AF was raised. This indicates that there is a strong interaction between 3A5AF and water molecules, and the high polarity of 3A5AF disrupts the hydrogen bond network of H_2_O molecules [33]. This can be further confirmed by nuclear magnetic resonance spectroscopy, as shown in Figure 3h. When 3A5AF is introduced, the ^1^H NMR shows a low field shift, which is the result of the formation of a hydrogen bond between a proton in a water molecule and two highly electronegative oxygen atoms. This, in turn, disrupts the initial hydrogen bond network of H_2_O and further reduces unnecessary side reactions [34].

Raman spectroscopy was further utilized to reveal the changes in the hydrogen bond network in the electrolyte. As shown in Figure 3i, the broad O─H stretching envelope between 3000 and 3800 cm^−^^1^ was deconvoluted into two components: strong hydrogen bonds at 3220 cm^−^^1^ and weak hydrogen bonds at 3405 cm^−^^1^ [35]. The introduction of 3A5AF markedly altered the hydrogen‐bonding environment: the population of strong hydrogen bonds increased (from 47.43% to 60.65%), whereas that of weak hydrogen bonds diminished significantly. This is because there is a strong interaction between 3A5AF and H_2_O, which forms a stable intermolecular hydrogen bond. This interaction disrupts the original hydrogen bonds between water molecules, reducing the content of free water molecules and effectively inhibiting the occurrence of HER [35].

**FIGURE 4:**
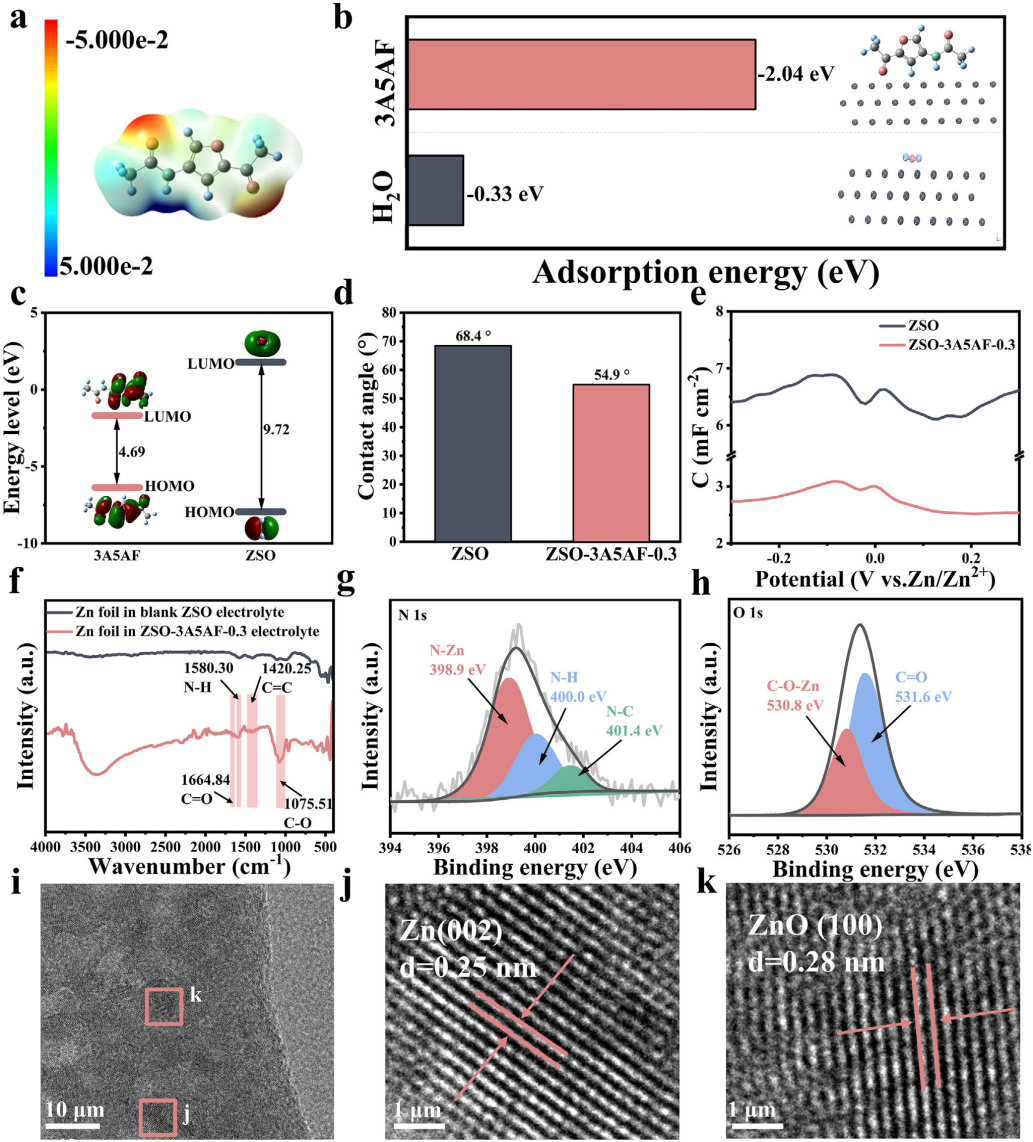
Investigation of 3A5AF's stable adsorption on the surface of zinc anode. (a) Molecule electrostatic potential map of 3A5AF. (b) A comparison of the adsorption energies between H_2_O and 3A5AF molecules on the Zn(002) surface. (c) The LUMO and HOMO energy levels of the H_2_O molecule and the 3A5AF molecule. (d) Contact angles for the blank ZSO and ZSO‐3A5AF‐0.3 electrolytes on the zinc anode surface. (e) Double‐layer capacitance of the Zn||Zn symmetric battery based on blank ZSO and ZSO‐3A5AF‐0.3 electrolyte. (f) The ATR‐FTIR spectra of the zinc foil after being cycled 20 cycles in the blank ZSO and ZSO‐3A5AF‐0.3 electrolytes, respectively. (g) N 1s and (h) O 1s core‐level spectra of the zinc foil cycled in the ZSO‐3A5AF‐0.3 electrolyte. (i–k) HRTEM image of Zn anode after cycling in ZSO‐3A5AF‐0.3 electrolyte.

In parallel to its role in solvation structure regulation, 3A5AF exhibits a strong tendency to adsorb onto the zinc anode, facilitating its incorporation into the evolving SEI layer. First, the electrostatic potential on the surface of the 3A5AF molecule was calculated by DFT, as shown in Figure 4a. The negative charges are mainly concentrated on the oxygen functional groups in the molecule. These polar groups will act as strong zinc‐philic sites, which indicates that there will be a potential competitive relationship between 3A5AF and water molecules during the adsorption process on the zinc surface [1]. Therefore, in order to reveal the adsorption of 3A5AF, the adsorption energies of H_2_O and 3A5AF on the Zn(002) crystal plane were calculated as shown in Figure 4b. Compared with the blank ZSO (−0.33 eV), the adsorption energy of 3A5AF on the Zn(002) crystal plane is significantly more negative (−2.04 eV), indicating that 3A5AF preferentially adsorbs on the zinc surface during the electroplating process. The strong adsorption effect is conducive to participating in the formation of the SEI during the zinc deposition process [36]. To further demonstrate the stronger binding ability between the 3A5AF molecule and the Zn surface, theoretical calculations were conducted at the molecular orbital level, as shown in Figure 4c. The highest occupied molecular orbital (HOMO) energy level of the 3A5AF molecule is significantly higher than that of the H_2_O molecule (−6.09 eV compared to −8.14 eV), indicating that the 3A5AF molecule has a stronger electron supply capacity. When the 3A5AF molecule comes into contact with the Zn negative electrode surface, its HOMO orbital is more inclined to interact with the empty orbitals on the Zn surface, thereby forming a stronger adsorption bond. This interaction enables the 3A5AF molecule to adsorb preferentially on the Zn negative electrode surface over water molecules [31]. In addition, the energy gap between the HOMO and LOMO of 3A5AF is narrower than that of H_2_O. This electronic feature is instrumental in optimizing the interfacial charge transfer and adsorption, which collectively contribute to the superior plating behavior of zinc [37].

Further experimental evidence confirmed the adsorption of 3A5AF onto the zinc anode and its participation in building the protective SEI layer. First, the contact angles (CA) of the blank ZSO and 3A5AF on the zinc metal surface were tested, as shown in Figure 4d. The contact angle of the electrolyte solution with 3A5AF added on the zinc anode surface was 13.5°, less than that of ZSO, indicating that its adsorption effect enhanced the surface wettability [28]. After standing for 1 min (Figure S6), the contact angle of the blank ZSO changed by 1.6°, while the contact angle of the electrolyte with 3A5AF was changed by 2.8°, further proving that 3A5AF was adsorbed on the zinc anode surface and thus improved the wetting performance of the electrolyte. The results of differential capacitance testing are shown in Figure 4e. Compared with the blank ZSO electrolyte, the capacitance decreased significantly after adding 3A5AF, indicating that the 3A5AF additive had firmly adhered to the zinc electrode surface and formed a thicker SEI layer [4]. To determine whether the 3A5AF molecules participated in the formation of the SEI film on the zinc anode surface, further characterization was conducted on the Zn anode after 20 cycles using ATR‐FTIR and XPS spectroscopy. The ATR‐FTIR spectrum (Figure 4f) of the anode from the ZSO‐3A5AF‐0.3 electrolyte displayed characteristic peaks (e.g., C═O, N─H) absent in the blank sample, indicating the participation of the additive [36, 38]. XPS analysis further confirmed this: the full survey (Figure S7a) detected C and N elements exclusive to 3A5AF. More critically, the high‐resolution C 1s spectrum (Figure S7b) revealed bonds specific to the 3A5AF molecule (C═O, C─O, C─N), while the N 1s (Figure 4g) and O 1s spectra (Figure 4h) showed Zn─N and C─O─Zn bonds [7]. This collective evidence unequivocally demonstrates a stable chemical adsorption of 3A5AF on the zinc anode and its role in forming a protective SEI film [39]. Thus, it effectively prevents the direct contact between zinc metal and water molecules, inhibits the corrosion of the zinc anode, and the hydrogen evolution reaction.

The components of the SEI on the zinc anode surface were further investigated by TEM‐EDS analysis. (HRTEM). The elemental mapping (Figure S8) showed a uniform distribution of Zn, S, C, O, and N on the cycled zinc anode surface in the ZSO‐3A5AF‐0.3 electrolyte, which also proves the existence of the SEI layer produced from the 3A5AF. More importantly, the Zn anode after cycling in ZSO‐3A5AF‐0.3 electrolyte, lattice fringes corresponding to Zn (002) and ZnO (100) were identified in the high‐resolution TEM images as shown in Figure 2j–k, indicating that ZnO is one of the constituents of the SEI film, and zinc‐ion deposition mainly follows 3D diffusion on the Zn (002) crystal plane, which is beneficial for enhancing the long‐cycle life of zinc‐ion batteries [40]. In contrast, the high‐resolution TEM images and EDS mapping of the Zn anode cycling in blank ZSO electrolyte (Figure S9) mainly showed lattice fringes of Zn (101) and Zn (100), with no formation of an SEI film. This suggests active surface causes the zinc anode to be directly exposed to the electrolyte during cycling, leading to continuous, uncontrollable side reactions. All of these results support the positive role of this additive in constructing a dense and continuous SEI layer on the zinc anode [41].

**FIGURE 5:**
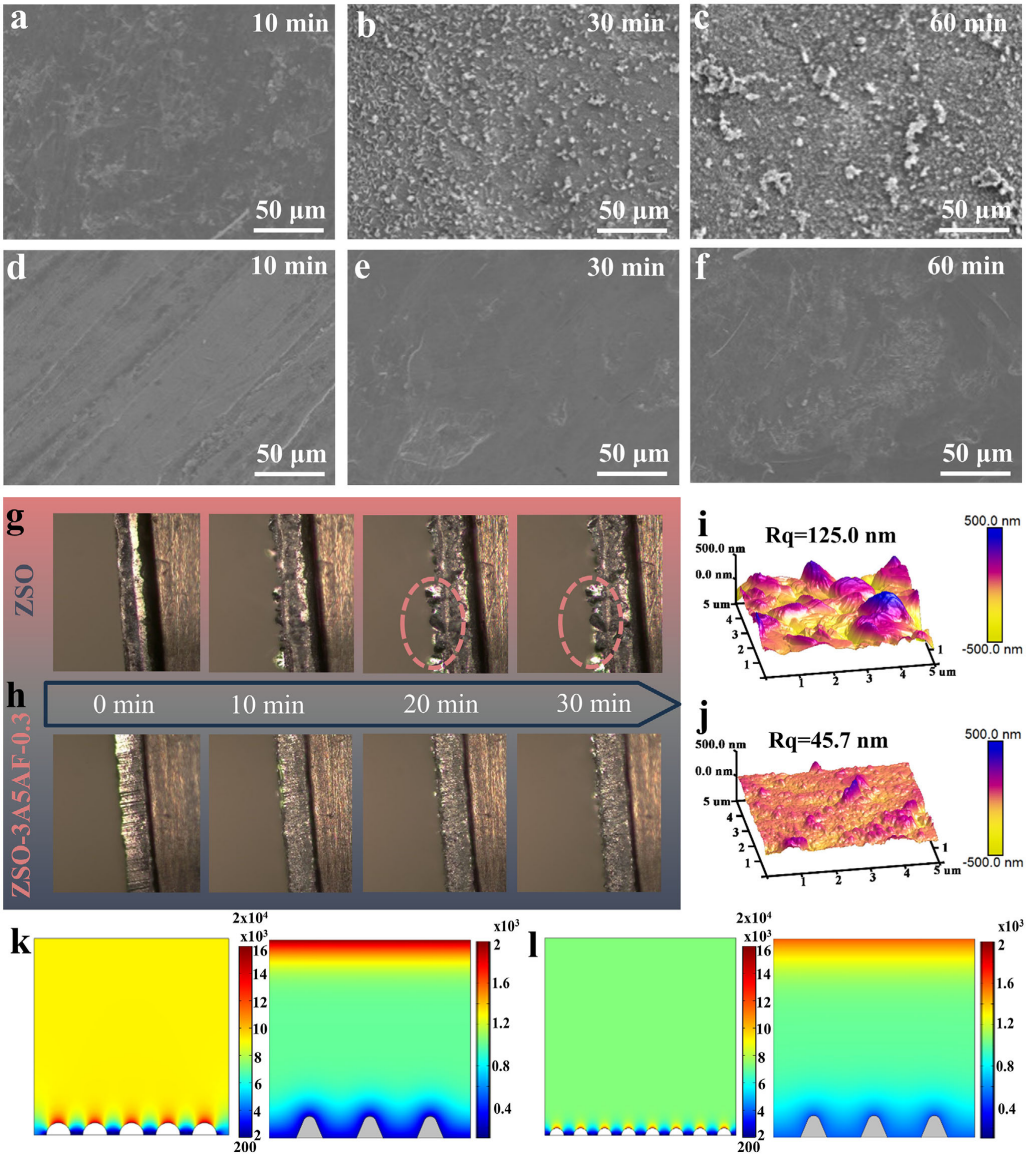
Roles of 3A5AF in uniformly depositing zinc ions and inhibiting dendrite growth. SEM images of zinc foil depositing in blank ZSO for (a) 10 min, (b) 30 min, and (c) 60 min; SEM images of zinc foil depositing in electrolyte with 3A5AF for (d) 10 min, (e) 30 min, and (f) 60 min. In situ optical microscopic images of Zn foil in (g) blank ZSO and (h) ZSO‐3A5AF‐0.3 under an electric current density of 5 mA cm^−2^. AFM images of the zinc anode after 50 cycles in (i) blank ZSO and (j) ZSO‐3A5AF‐0.3 electrolyte. The electric field distribution and ion flux at the interface between Zn anode and (k) blank ZSO electrolyte and (l) ZSO‐3A5AF‐0.3 as simulated by COMSOL.

The dual functions of 3A5AF additive, namely the restructuring of the solvation structure of the electrolyte and the protection of the negative electrode interface, are expected to simultaneously solve the failure problem of zinc negative electrode from both the solution phase and the electrode interface dimensions. Thus, it can play a positive role in inducing uniform Zn deposition and inhibiting dendrite growth. To verify this positive effect, first, we statistically observed the morphological changes on the surface of zinc negative electrode under different deposition times using scanning electron microscopy (SEM). As shown in Figure 5a–c, after deposition for 10 min in the blank ZSO, no significant changes were observed on the surface of the zinc foil. However, over time, dendrites gradually formed on the surface of the zinc foil, and by 30 min, the surface began to exhibit uneven morphology, accompanied by the formation of protrusions. Until 60 min, various protruding dendrites and white secondary product aggregates could be clearly observed. Conversely, the use of the 3A5AF‐supplemented electrolyte effectively maintained a smooth zinc surface with no detectable dendrites during the first 30 min of deposition, as evidenced in Figure 5d–f. Even after 60 min of deposition, the zinc foil surface remained uniformly covered with fine particles. To present the contrast effect more clearly, this uniform deposition was further confirmed using an in situ optical microscope at a current density of 5 mA cm^−2^. As shown in Figure 5g, 10 min after deposition, irregular zinc protrusions appeared on the surface of the zinc negative electrode, and these protrusions became larger after 30 min. In contrast, the zinc negative electrode in the ZSO‐3A5AF‐0.3 electrolyte always maintained uniform and dense Zn deposition without obvious protrusions, indicating that the 3A5AF additive enhanced the inhibition of dendrites, making the interface environment of the zinc negative electrode more stable (Figure 5h) [24].

Second, the 3D morphology after fifty times deposition cycles under a current density of 1 mA cm^−^^2^ was observed using atomic force microscopy (AFM), as shown in Figure 5i,j. In the blank ZSO electrolyte, the surface of the zinc negative electrode presented a significantly rough state, with a surface height of 125.0 nm, and the surface was prone to short circuit in traditional electrolytes; in contrast, in the zinc negative electrode with ZSO‐3A5AF‐0.3 electrolyte, it was smoother, with a surface height of only 45.7 nm. This comparative result further proved that the introduction of 3A5AF as an additive could significantly improve the uniformity of Zn deposition and effectively inhibit dendrite growth, thereby improving the electrochemical performance of the zinc negative electrode [42].

Third, the electric field and Zn^2^^+^ concentration gradients in the blank ZSO electrolyte and ZSO‐3A5AF‐0.3 electrolyte were simulated using the finite element method. Due to the poor affinity of zinc itself, zinc ions tend to randomly deposit on the surface of the zinc negative electrode, thereby forming dendritic protrusion structures. The formation process of these dendrites significantly enhances the electric field intensity around them, causing Zn^2^^+^ ions to rapidly accumulate at these points during diffusion, thereby further exacerbating the uneven distribution of Zn ion concentration [43]. This led to the formation and continuous growth of zinc dendrites, as shown in Figure 5k. Conversely, in the electrolyte containing 3A5AF, due to the aggregation of 3A5AF at the protruding tips of the zinc metal surface, an electrostatic shielding layer is formed, which helps to uniformly distribute the electric field, resulting in a significant reduction in the electric field intensity at the protrusion points. This phenomenon promotes the deposition of Zn^2^^+^ ions in the adjacent areas of the negative electrode, leading to a more uniform surface concentration distribution and reducing the possibility of dendrite formation, as shown in Figure 5l. This is conducive to achieving high reversibility of the zinc electroplating and peeling process [27, 44].

Finally, the regulatory mechanism of 3A5AF on the nucleation and growth behavior of Zn was investigated by electrochemical characterization. A reduction in nucleation overpotential from 45.46 mV (ZSO, |bb’|) to 42.63 mV (ZSO‐3A5AF‐0.3, |aa’|) was observed in the CV measurements of the symmetric cell (Figure S10), suggesting that the 3A5AF electrolyte system has higher Zn deposition kinetics and a lower nucleation barrier [38]. The chronoamperometry (CA) test under a constant overpotential of 150 mV showed (Figure S11) that in the initial nucleation stage, the CA curve exhibited a trend of rapid current increase within the first 10 s, corresponding to the Zn^2+^ nucleation process. As the deposition continued, the current density of the blank ZSO electrolyte significantly increased. This was mainly due to the 2D diffusion of Zn^2+^ and the formation of zinc dendrites, resulting in a loose and rough electrode morphology [45]. In contrast, in the 3A5AF electrolyte, the current density slightly increased, and it gradually stabilized with the deposition time, indicating that the 2D diffusion of Zn^2+^ was inhibited and the growth of zinc dendrites was reduced. This optimization can be attributed to the electrostatic shielding effect of 3A5AF. Specifically, 3A5AF adsorbed on the zinc electrode prevented the lateral diffusion of Zn^2+^ and the aggregation into dendrites, thereby forming a uniform and dense zinc deposition morphology [27].

The excellent uniform deposition behavior is also highly dependent on the transport kinetics of Zn^2+^. Therefore, the ionic conductivity of the electrolytes was examined. Measurement of the ionic conductivity (Figure S12) revealed an increase from 49.8 mS cm^−^^1^ for the blank ZSO to 56.5 mS cm^−^^1^ for the ZSO‐3A5AF‐0.3 electrolyte. Higher ionic conductivity implies a faster rate of ionic transmission [3]. Then, the impedance changes of Zn||Zn symmetric cells before and after polarization in blank ZSO and ZSO‐3A5AF‐0.3 electrolytes were tested to calculate t_Zn_2+. As shown in Figures S13 and S14, in the blank ZSO sample, the number of t_Zn_2+ only reached 0.28. After adding 3A5AF, the migration number of Zn^2+^ significantly increased to 0.60. This change is largely attributed to strong 3A5AF‐water interactions within the zinc ion's primary solvation shell, which accelerates the desolvation kinetics of Zn^2+^ from the [Zn(H_2_O)_6_]^2+^ complex. Eventually, it increases the transfer number of Zn^2+^, facilitating the uniform distribution of Zn^2+^ flux and promoting the dendrite‐free zinc deposition [42].

**FIGURE 6:**
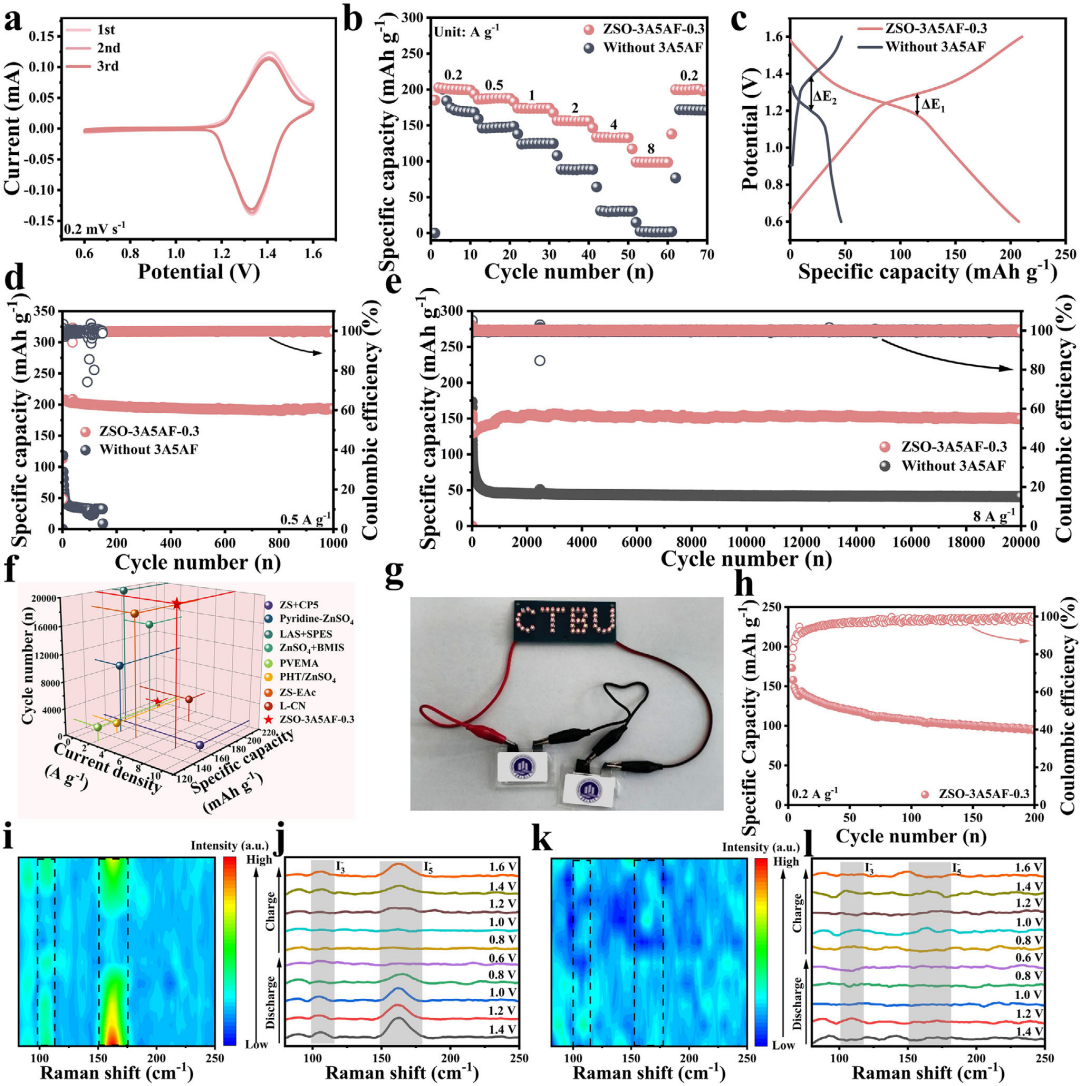
The electrochemical performances of Zn‐I_2_ full cells. (a) The CV curve of the Zn||I_2_ full‐cell with ZSO‐3A5AF‐0.3 at a scanning rate of 0.2 mV s^−1^. (b) The rate performance of the Zn||I_2_ full battery in ZSO and ZSO‐3A5AF‐0.3 electrolytes. (c) The constant current charge–discharge curve of the Zn||I_2_ full cell at a current density of 0.5 A g^−1^. The cycling performances of Zn||I_2_ full cell at (d) low current of 0.5 A g^−1^ and (e) high current of 0.5 A g^−1^ in ZSO and ZSO‐3A5AF‐0.3 electrolytes. (f) Comparison of electrochemical performance of Zn||I_2_ full cell. (g) A digital photo of two soft‐pack batteries lighting up “CTBU” LED lamp. (h) Galvanostatic cycling of the Zn||I_2_ pouch cell at 0.2 A g^−^^1^ in ZSO‐3A5AF‐0.3 electrolyte. In situ Raman spectrum of Zn||I_2_ battery assembled in (i,j) ZSO system and (k,l) ZSO‐3A5AF‐0.3 system.

Finally, a Zn||I_2_ full‐cell was assembled to verify the feasibility of 3A5AF in practical applications. The CV test indicated that within the voltage range of 0.6 – 1.6 V (vs. Zn^2+^/Zn), there was only one pair of I_2_/I^−^ conversion reduction‐oxidation peaks (1.33/1.42 V), proving that the electrolyte additive was stable within this working voltage range and did not undergo redox reactions, contributing capacity [9]. Additionally, from Figure 6a, it can be observed that the CV cycle curves in the first three cycles of I_2_/I^−^ oxidation‐reduction peak pairs exhibited high reproducibility, indicating good reversibility. The rate capability of Zn||I_2_ full cells employing blank ZSO and ZSO‐3A5AF‐0.3 electrolytes was systematically evaluated over current densities ranging from 0.2 to 8 A g^−^^1^. As summarized in Figure 6b, the full cell incorporating ZSO‐3A5AF‐0.3 maintained clearly superior discharge capacities relative to the blank ZSO system across all tested rates, delivering 202.5, 186.5, 174.2, 156.8, 133.6, and 99.0 mAh g^−^^1^ at 0.2, 0.5, 1, 2, 4, and 8 A g^−^^1^, respectively. Furthermore, upon reverting the current density to 0.2 A g^−^^1^, the discharge capacity promptly recovered to 199.7 mAh g^−^^1^, underscoring the exceptional reversibility imparted by the 3A5AF additive. Moreover, the GCD curve (Figure 6c) also showed that the Zn||I_2_ full‐cell with ZSO‐3A5AF‐0.3 electrolyte had a longer discharge plateau and smaller polarization (ΔE_1_ < ΔE_2_). The ultra‐long discharge plateau of ZSO‐3A5AF‐0.3 indicates that the electrode can quickly achieve a complete oxidation‐reduction reaction, promoting the rapid conversion of I_2_/I^−^, and thereby obtaining a higher capacity [12]. The enhanced reaction kinetics afforded by the 3A5AF additive were further quantified by EIS (Figure S15). The Nyquist plots exhibit a semicircle in the high‐frequency region, representing the charge‐transfer resistance (*R*_ct_). The significantly smaller diameter of this semicircle for the full cell with ZSO‐3A5AF‐0.3 indicates a substantially lower Rct than that of the blank ZSO‐based full cell [39].

To more comprehensively evaluate the cycling performance of the Zn‐I_2_ full‐cell based on ZSO‐3A5AF‐0.3 electrolyte, a long‐cycle test was conducted. The cycling stability at 0.5 A g^−^^1^ differed markedly between the two electrolytes as shown in Figure 6d,e. While the blank ZSO‐based cell exhibited rapid capacity fade and unstable efficiency within 100 cycles, the ZSO‐3A5AF‐0.3 cell maintained excellent stability, commencing with a high initial discharge capacity of 207.2 mAh g^−^^1^. More importantly, when the current density was increased to 8 A g^−1^, the effect of 3A5AF was still significant, enabling the battery to maintain 20 000 cycles, and the capacity retention rate of the full battery from 26.9% increased to 93.9% after cycling. Compared with many additives previously reported, 3A5AF undoubtedly shows extremely prominent advantages (Figure 6f; Table S2). Furthermore, we also assembled a Zn||I_2_ pouch battery (2 × 3 cm^2^) to demonstrate the practical potential of the 3A5AF electrolyte. This pouch battery was able to supply normal light emission for the LED marked with CTBU, as shown in Figure 6g. Moreover, at a current density of 0.2 A g^−1^, the Zn||I_2_ pouch battery was able to work for more than 200 times, maintaining a reversible capacity of 94.1 mAh g^−1^ (Figure 6h), indicating good compatibility with the I_2_ cathode and the versatility of the optimized electrolyte.

The shuttle effect of polyiodide ions is a key bottleneck restricting the development of Zn‐I_2_ batteries. The loss of active substances and the side reactions of the zinc anode caused by it seriously limit the battery performance. Therefore, finally in situ Raman spectroscopy confirmed that the 3A5AF additive can effectively inhibit the shuttling of polyiodide ions in the actual Zn‐I_2_ battery system. As shown in Figure 6i,j, in the Zn‐I_2_ cells using ZSO electrolyte, distinct I_3_^−^ and I_5_^−^ characteristic peaks at 110 and 165 cm^−^^1^, respectively, were detected at the anode interface. Moreover, after the charging process is completed, these polyiodide signals still persist, indicating that their oxidation reaction kinetics are sluggish and that efficient reversible redox conversion has not been achieved. This irreversible process will lead to the continuous loss and capacity attenuation of the positive electrode iodine active material. In the system using ZSO‐3A5AF‐0.3 electrolyte (Figure 6k,I), no significant polyiodide ion characteristic peaks were detected at the anode interface throughout the charging and discharging process. This direct contrast strongly demonstrates that the 3A5AF additive can effectively anchor polyiodide ions and inhibit their shuttle to the zinc anode, thereby providing a key guarantee for enhancing the cycle reversibility of Zn‐I_2_ batteries [8, 46].

## 3 Conclusions

In conclusion, we have successfully developed a biomass‐derived additive, 3‐acetylamino‐5‐acetylfuran (3A5AF), enriched with polar N/O functional groups, which significantly enhances the Coulombic efficiency and cycling stability of Zn anodes at an ultralow concentration (0.3 mg mL^−^^1^). Theoretical calculations and experimental results collectively demonstrate that 3A5AF molecules participate in the solvation structure of hydrated Zn^2^^+^, effectively weakening Zn^2^^+^–H_2_O interactions and reducing the activity of water molecules in the solvation sheath. Moreover, 3A5AF adsorbs strongly on the Zn anode surface, limiting water access and thereby suppressing corrosion and rampant 2D diffusion, leading to uniform and dense Zn deposition. As a result, exceptional electrochemical performance is observed: the Zn||Zn symmetric cell achieves a cycle life of 2700 h at 1 mA cm^−^^2^ and 1 mAh cm^−^^2^, and the Zn||Cu configuration notably yields an average CE of 99.51% over 800 cycles, further confirming the excellent reversibility of zinc deposition. Furthermore, the assembled Zn||I_2_ full cell demonstrates enhanced cycling performance, retaining a reversible capacity of 192.6 mAh g^−^^1^ after 1000 cycles at 0.5 A g^−^^1^. Even at a high current density of 8 A g^−^^1^, the cell maintains a high capacity of 149.6 mAh g^−^^1^ over 20 000 cycles. This work offers a green, economical, and effective strategy for developing high‐performance aqueous Zn‐I_2_ batteries.

## 4 Experimental

### 4.1 Preparation and Purification of 3A5AF

According to our previous report [47], 880 mg NAG and 340 mg LiCl were added in 40 mL N‐methylpyrrolidone and stirred at 50°C for 10 min to ensure complete dissolution. The resulting solution was transferred into a water bath reactor, and then the reactor was sealed and heated in an oil bath at 200°C for 1 h. Once the reaction mixture reached room temperature, suction filtration was performed to isolate the crude 3A5AF solution.

Next, 100 mL of the crude 3A5AF reaction solution was mixed with 100 mL of deionized water. This diluted solution was then subjected to extraction using ethyl acetate (EA). The extracted EA phase was collected, and saturated NaCl was added and concentrated in a rotary evaporator at 45°C to obtain the crude solid product of 3A5AF. The final purification of the crude product was achieved using silica gel column chromatography, with ethyl acetate employed as the eluent, yielding pure 3A5AF.

### 4.2 Electrolyte Preparation

Dissolve 5.7512 g of ZnSO_4_·7H_2_O in 10 mL of deionized water and denote it as ZSO. Subsequently, add different amounts of 3A5AF (0.01, 0.03, and 0.05 g) respectively into 2 m ZnSO_4_ electrolyte to prepare ZnSO_4_ + 3A5AF electrolytes of different concentrations, and label them as ZSO‐3A5AF‐0.1, ZSO‐3A5AF‐0.3, and ZSO‐3A5AF‐0.5, respectively.

### 4.3 Electrode Preparation

First, the I_2_@CNT composite was synthesized by mixing CNT and I_2_ powder (1:1 in mass ratio) and heating at 90°C for 4 h in the hydrothermal reactor. Then, according to the mass ratio of 7:2:1, weigh the I_2_@CNT powder, conductive carbon black, and polyvinylidene fluoride (PVDF), and add an appropriate amount of N‐methylpyrrolidone (NMP) to prepare the electrode slurry. After the slurry becoming homogenously, it was applied onto the stainless steel foil and dried under a vacuum oven at 60°C for 12 h. Ultimately, the electrodes were cut into circular pieces with a diameter of 12 mm to serve as the I_2_ positive electrode sheets.

### 4.4 Materials Characterizations

The changes in hydrogen bonds in various electrolytes were investigated using attenuated total reflection Fourier transform infrared spectroscopy (ATR‐FTIR, Thermo Fisher Scientific Nicolet iS20, USA), Raman spectrometer (Renishaw Qontor, England), and Bruker‐Avance III HD (Germany) nuclear magnetic resonance spectroscopy (NMR, 400 MHz). The contact angles between zinc metal and various electrolytes were measured using a contact angle goniometer (MY‐SPCAX3, Shanghai Weichuan Precision Instrument Co., Ltd). The surface composition of the Zn anode after cycling was investigated by Thermo Fisher ESCALAB Xi^+^ (USA) X‐ray photoelectron spectroscopy (XPS). The surface morphology of zinc metal under different operating conditions was characterized using a scanning electron microscope (SEM, Hitachi SU‐8010). The transmission electron microscope (TEM) images were captured with JEOL JEM‐F200. In situ microscopic images of the zinc deposition process were monitored using an optical microscope (YD650). The in situ Raman spectra was tested on the RENISHAW in Via Qontor machine. Atomic force microscopy (AFM, Bruker DimensonICON, United States) was used to record the atomic force microscopy images of the zinc negative electrode after cycling under different electrolytes. The pH values of various electrolytes were measured using a pH meter (PHS‐3E). The electrolytes’ ionic conductivities were determined by an ionic conductivity meter (DDS‐11A). The rotational viscosity of various electrolytes was tested with a rotational viscometer (NDJ‐1S).

### 4.5 Electrochemical Test

Four types of cells—Zn||Zn symmetric, Zn||Cu asymmetric, Zn||Ti asymmetric, and Zn||I_2_ full‐cells—were assembled in CR2032 coin‐type configurations, with a consistent electrolyte volume of 80 µL used for each cell. The cycling and rate capabilities as well as CE of batteries were performed on LAND battery system. The chronoamperometry (CA), cyclic voltammetry (CV) curves, electrochemical impedance spectroscopy (EIS), Tafel curves, LSV curves and i‐t curves were performed on the CHI660e.

The Zn^2+^ transfer number (t_Zn_2+) is calculated using the following formula:

(1) $$t_{Zn^{2+}}=\frac{I_{s}\left(\Delta V-I_{0}R_{0}\right)}{I_{0}\left(\Delta V-I_{s}R_{s}\right)}\;$$

Here, ∆*V* represents the polarization voltage. The initial and steady‐state values are denoted by *I*_0_ and *R*_0_ for current and resistance, with *I*_s_ and *R*_s_ representing the respective steady‐state parameters.

### 4.6 Theoretical Calculations

The Gaussian 09 software package was utilized to perform quantum chemical calculations, such as those for the highest occupied molecular orbital (HOMO), the lowest unoccupied molecular orbital (LUMO), and van der Waals‐inclusive electrostatic potential (ESP). Initial geometry optimizations were carried out employing the B3LYP functional with the 6–311+G(d,p) basis set.

The adsorption energy calculations were performed using VASP [48] with the projector augmented wave (PAW) [49] pseudo‐potentials for describing the atomic cores interaction and valence electrons. The exchange‐correlation interactions were characterized using the Perdew–Burke–Ernzerhof (PBE) [50] functional within the generalized gradient approximation (GGA) [51], with a plane‐wave cut‐off energy defined as 520 eV and convergence criteria set to 0.05 eV Å^−^^1^ for forces and 10^−^^5^ eV for energy. A DFT‐D_3_ correction with Grimme scheme was used to account for the dispersion interaction [52]. A vacuum of 15 Å was set to avoid interaction along *z*‐axis of Zn (002). The Brillouin zone was sampled with the Gamma‐centered Monkhorst–Pack [53] scheme *k*‐point grid of 1×1×1 for geometry optimization.

The desolvation energies of [Zn(H_2_O)_6−x_]^2+^ and [Zn(H_2_O)_5−x_(3A5AF)]^2+^ (*x* = 1–5) are delineated as follows, respectively:

(2) $$\begin{array}{c} \Delta E=\left\{\mathrm{xE}\left(H_{2}O\right)+E\left\{{\left[\mathrm{Zn}{\left(H_{2}O\right)}_{6-x}\right]}^{2+}\right\}\right\}-E\left\{{\left[\mathrm{Zn}{\left(H_{2}O\right)}_{6}\right]}^{2+}\right\}\;\;\;\; \end{array}$$

(3) $$\begin{array}{ccc} \Delta E & = & \left\{\mathrm{xE}\left(H_{2}O\right)+E\left\{{\left[\mathrm{Zn}{\left(H_{2}O\right)}_{5-x}\left(3A5\mathrm{AF}\right)\right]}^{2+}\right\}\right\}\\ & & -\;E\left\{{\left[\mathrm{Zn}{\left(H_{2}O\right)}_{5}\left(3A5\mathrm{AF}\right)\right]}^{2+}\right\} \end{array}$$

### 4.7 Finite Element Simulation

To simulate the migration and deposition of zinc ions along with the distributions of electrochemical potential and current, a finite element model was constructed in COMSOL Multiphysics 6.2. This model utilized the secondary current distribution interface and accounted for the coupled electric and concentration fields. In the simplified 2D model battery, set the 50 µm and 50 µm square regions, and select the top volume region as the positive electrode and the bottom volume region as the negative electrode. Two different anode structures were prepared to characterize the effect of zinc ion deposition on these structures. The simulation setup specified an initial electrolyte ion concentration of 2.0 m, with the electrode surface potential set to 0.0 V. The electrode reaction kinetics were governed by the Butler–Volmer equation, employing an exchange current density of 100 A m^−^^2^ and a transfer coefficient of 0.5.

### 4.8 Molecular Dynamics Simulation

All MD simulations were performed under canonical ensemble conditions using the GROMACS package, along with the Amber ff14SB force field and the SPC water model [54]. With 120 3A5AF mole in the mixture of 1204 ZnSO_4_ and 33426 H_2_O (2 mol L^−1^), the container's length increased to 10 × 10 × 10 nm^3^. In each of the three directions, periodic boundary conditions were established. Electrostatic and van der Waals (vdW) interactions were handled with the PME and cutoff methods [55], respectively, with a uniform cutoff length of 1.0 nm applied to both. The charges of the restricted electrostatic potential (RESP) were employed in order to accurately simulate the electrostatic environment around the molecular surface. The time step was raised to 2 fs using the H‐angles restricting approach. A V‐rescale thermostat was used to regulate the temperature [56], and Berendsen barostat was used to regulate the pressure [57]. The system was first annealed (0–298 K) for equilibrium, followed by a 20 ns production simulation where the pressure coupling method was switched from Berendsen to Parrinello‐Rahman [58]. The resulting trajectories were analyzed by applying Equations (5) and (6) to compute the radial distribution function (RDF) and its coordination number (CN):

(4) $$\mathrm{RDF}\;\left(r\right)=g\;\left(r\right)=\frac{n\left(r\right)}{\rho 4\pi r^{2}\Delta r}\;$$

(5) $$\mathrm{CN}\;=\int_{0}^{r}\rho 4\pi r^{2}g\left(r\right)dr\;$$

where g(r) and CN are the associated RDF and coordination numbers, n(r) denotes the number of atoms located within a certain distance r around the central atom, and ρ is the total number density. The RDF and CN are calculated for the relevant data.

## Conflicts of Interest

The authors declare no conflicts of interest.

## Supporting information




**Supporting File**: advs73684‐0001‐SuppMat.docx.

## Data Availability

The data that support the findings of this study are available from the corresponding author upon reasonable request.
