# Supplementary material for: Regulating Zinc Anode Interface with an Environmental Biomass‐Derived Additive for Long‐Lifespan Aqueous Batteries
Source: Adv Sci (Weinh). 2026 Jan 7;13(16):e22511. doi: 10.1002/advs.202522511 (PMC13042373; doi:10.1002/advs.202522511)
Supplement: Supplementary file 1 — Supporting File: advs73684‐0001‐SuppMat.docx. [file ADVS-13-e22511-s001.docx]

**Supporting Information**

**Regulating Zinc Anode Interface with an Environmental Biomass-Derived Additive for Long-Lifespan Aqueous Batteries**

*Bingbo Ni^a,b,c^, Qian Wang^a,b^, Qiusheng Ma^a,d^, Junhui Cheng^a,e^, Xingxing Gu^b*^, Guangyin Li^a*^*

^a^ Northeast Institute of Geography and Agroecology, Chinese Academy of Sciences. Changchun 130102, China

^b^ Chongqing Key Laboratory of Environmental Catalysis, College of Environment and Resources, Chongqing Technology and Business University, Chongqing 400067, China

^c^ University of Chinese Academy of Sciences, Beijing 100049, China.

^d^ College of Hydrology and Water Resources, Hohai University, Nanjing 210098, China

^e^ School of Geographic and Oceanographic Science, Nanjing University, Xianlin Road 163, 210023 Nanjing, China

*^*^*Correspondences Email: x.gu@ctbu.edu.cn; liguangyin@iga.ac.cn


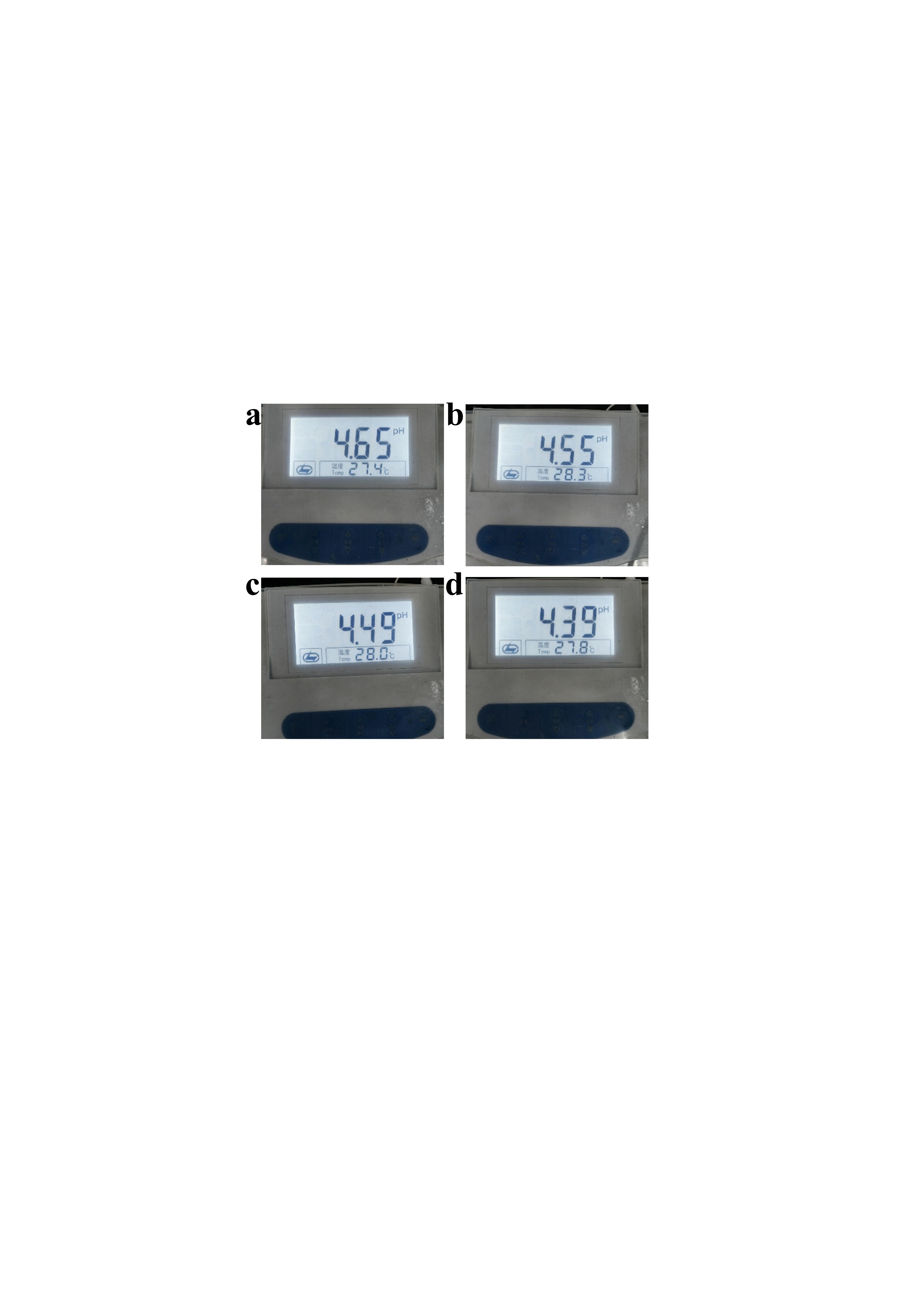


**Figure S1.** corresponding optical images for testing 2 M ZSO electrolyte containing different concentrations of 3A5AF additive.


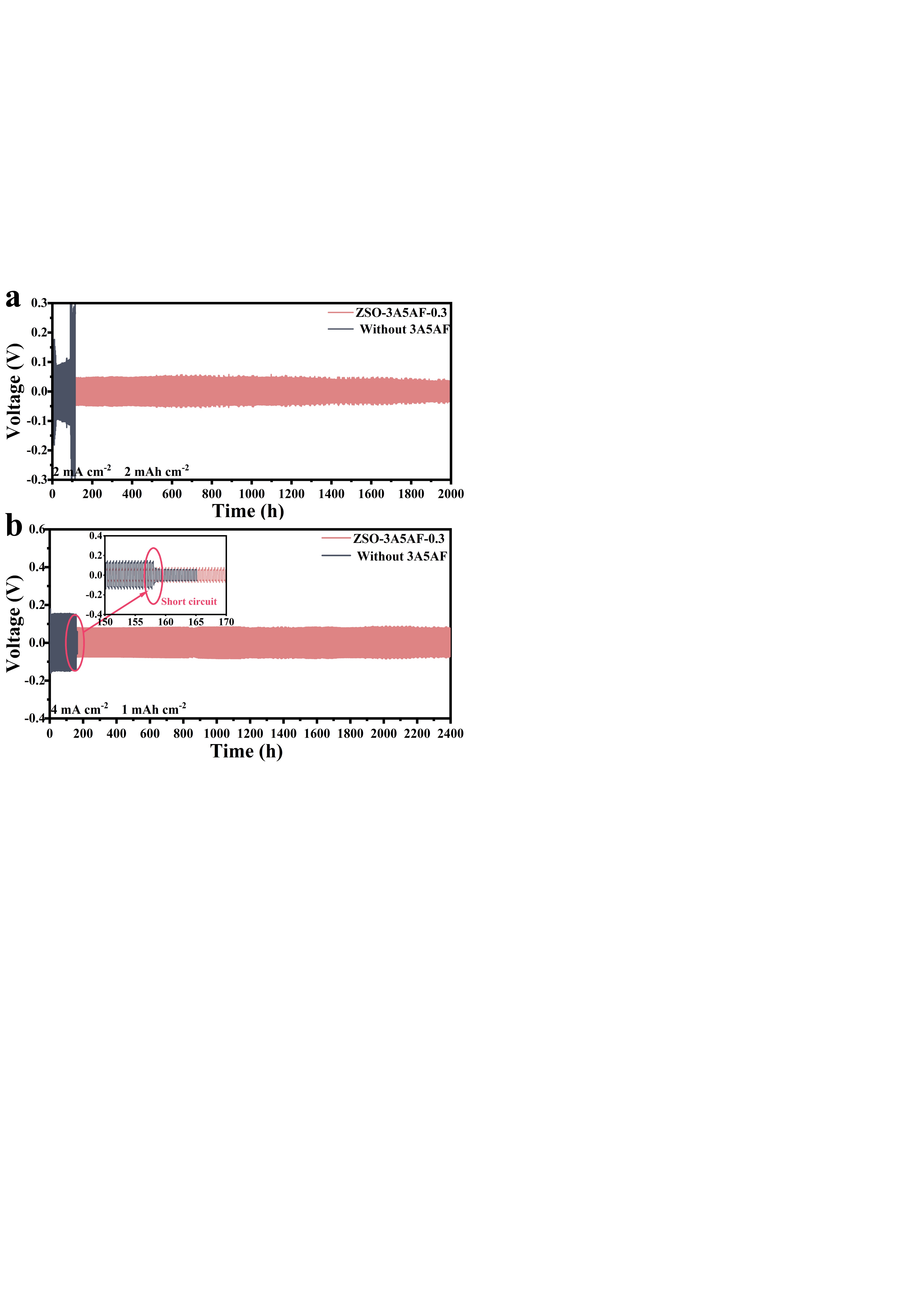


**Figure S2.** (a) Cycling performance of Zn||Zn symmetric cells using ZSO and ZSO-3A5AF-0.3 electrolytes at 2 mA cm^-2^ and 2 mAh cm^-2^. (b) Cycling performance of Zn||Zn symmetric cells using ZSO and ZSO-3A5AF-0.3 electrolytes at 4 mA cm^-2^ and 1 mAh cm^-2^.


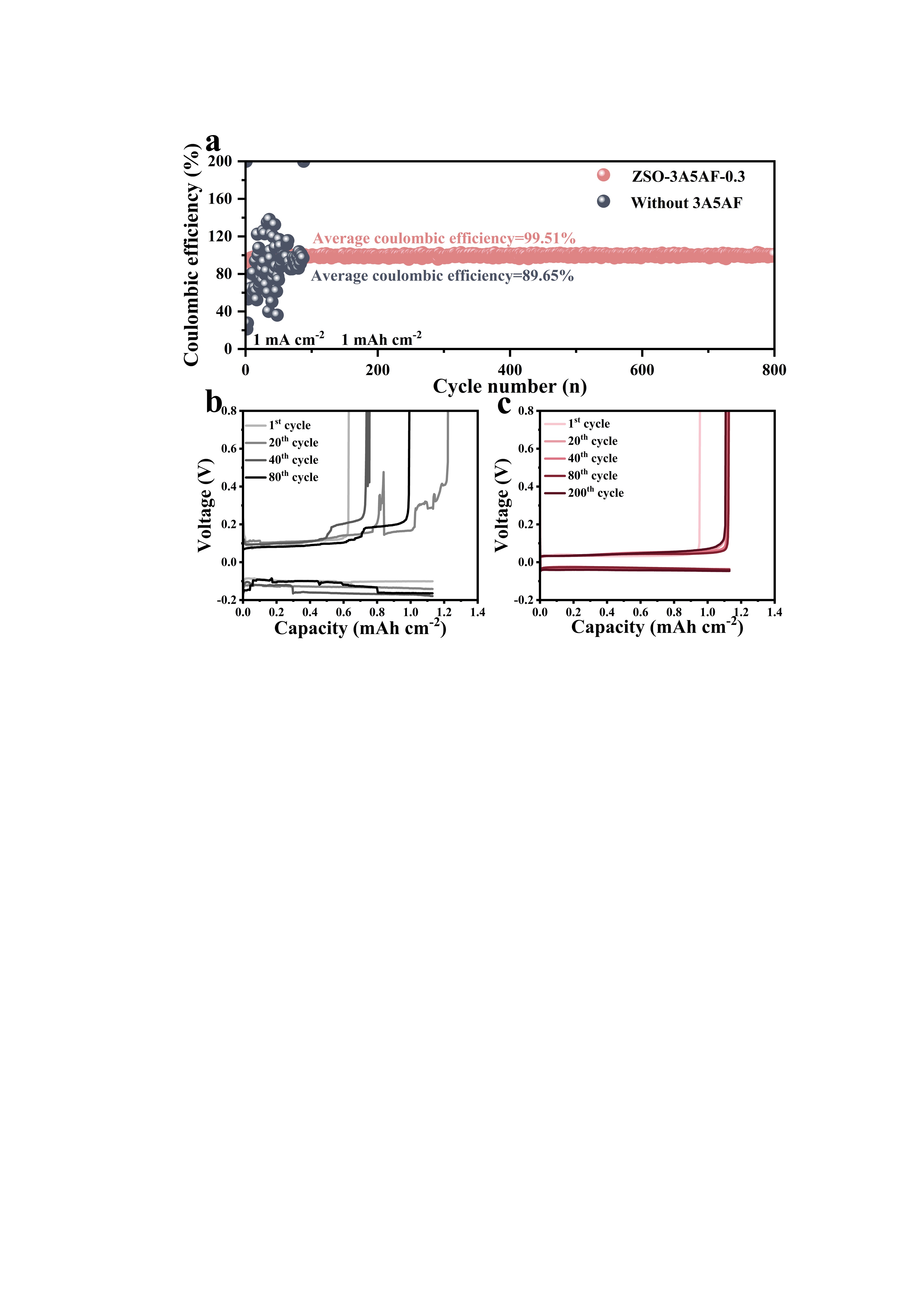


**Figure S3. (**a) CE of zinc plating/stripping in Zn||Cu batteries at 1 mA cm^-2^ and 1 mAh cm^-2^. Voltage capacity curves of Zn||Cu batteries with (b) blank ZSO and (c) ZSO-3A5AF-0.3 electrolytes.


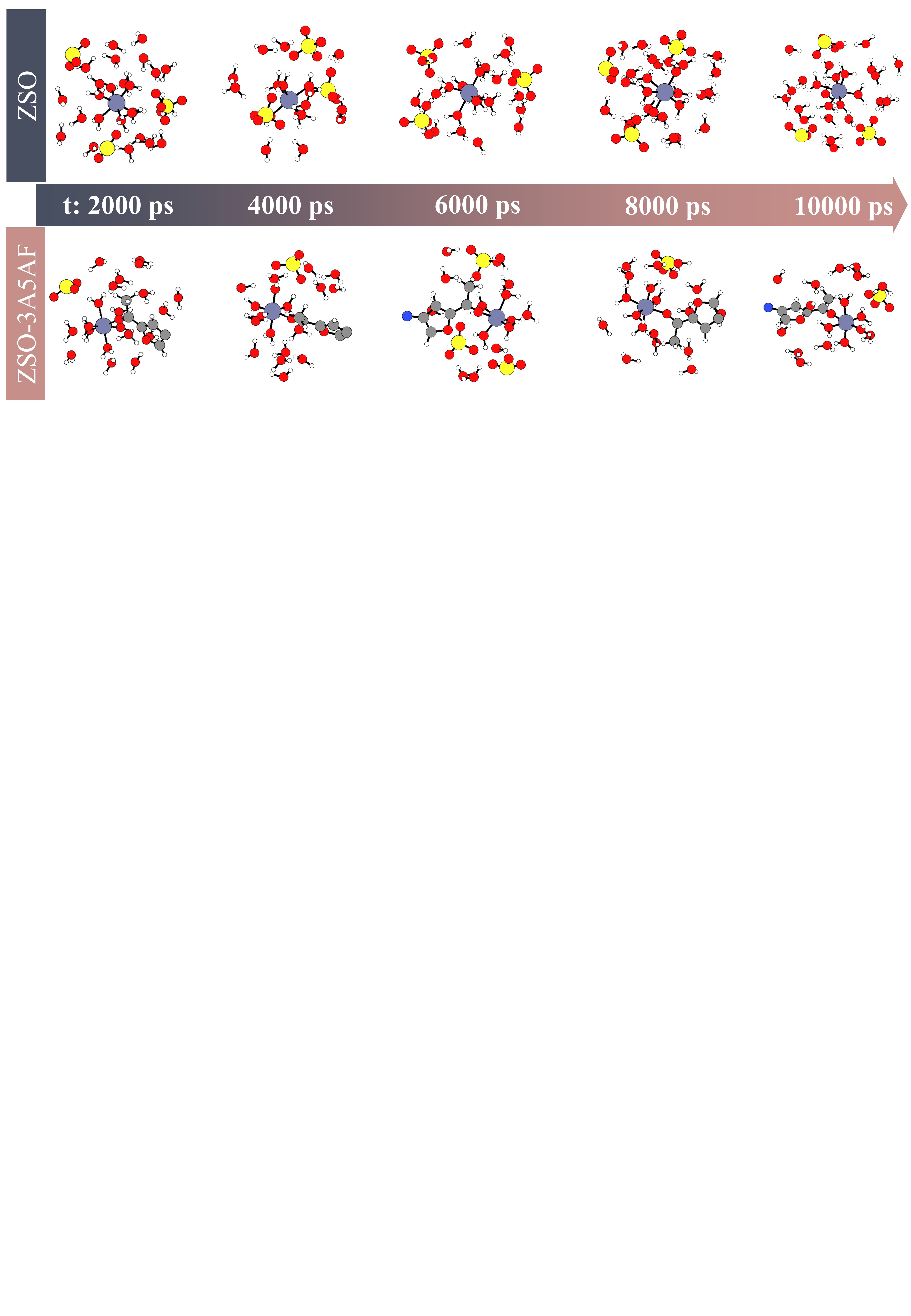


**Figure S4.** Dynamic MD simulation snapshot of the solvated sheath structure of blank ZSO and ZSO-3A5AF electrolytes.


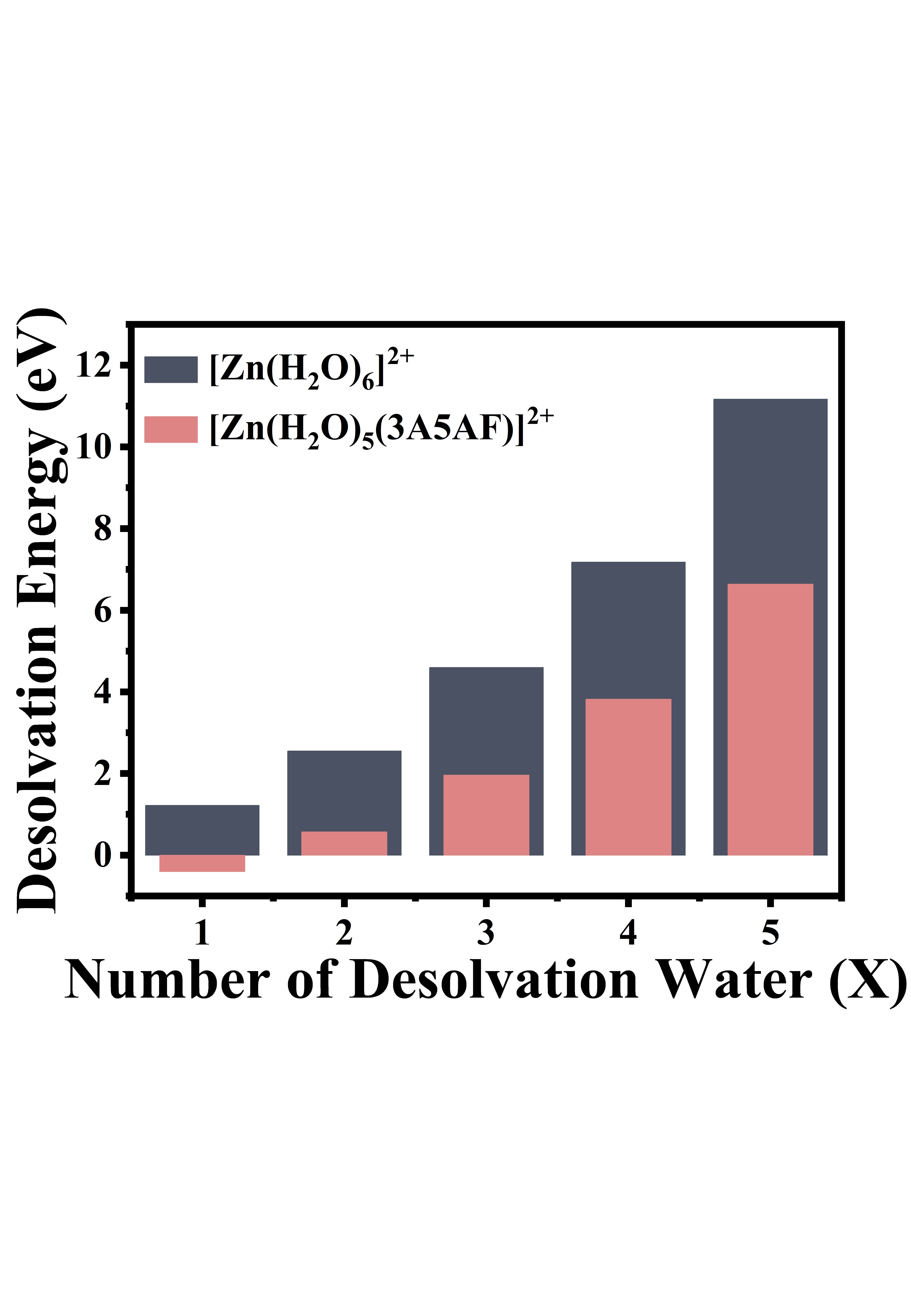


**Figure S5.** The desolvation energies of [Zn(H_2_O)_6-x_]^2+^ and [Zn(H_2_O)_5-x_(3A5AF)]^2+^ (x=1-5).


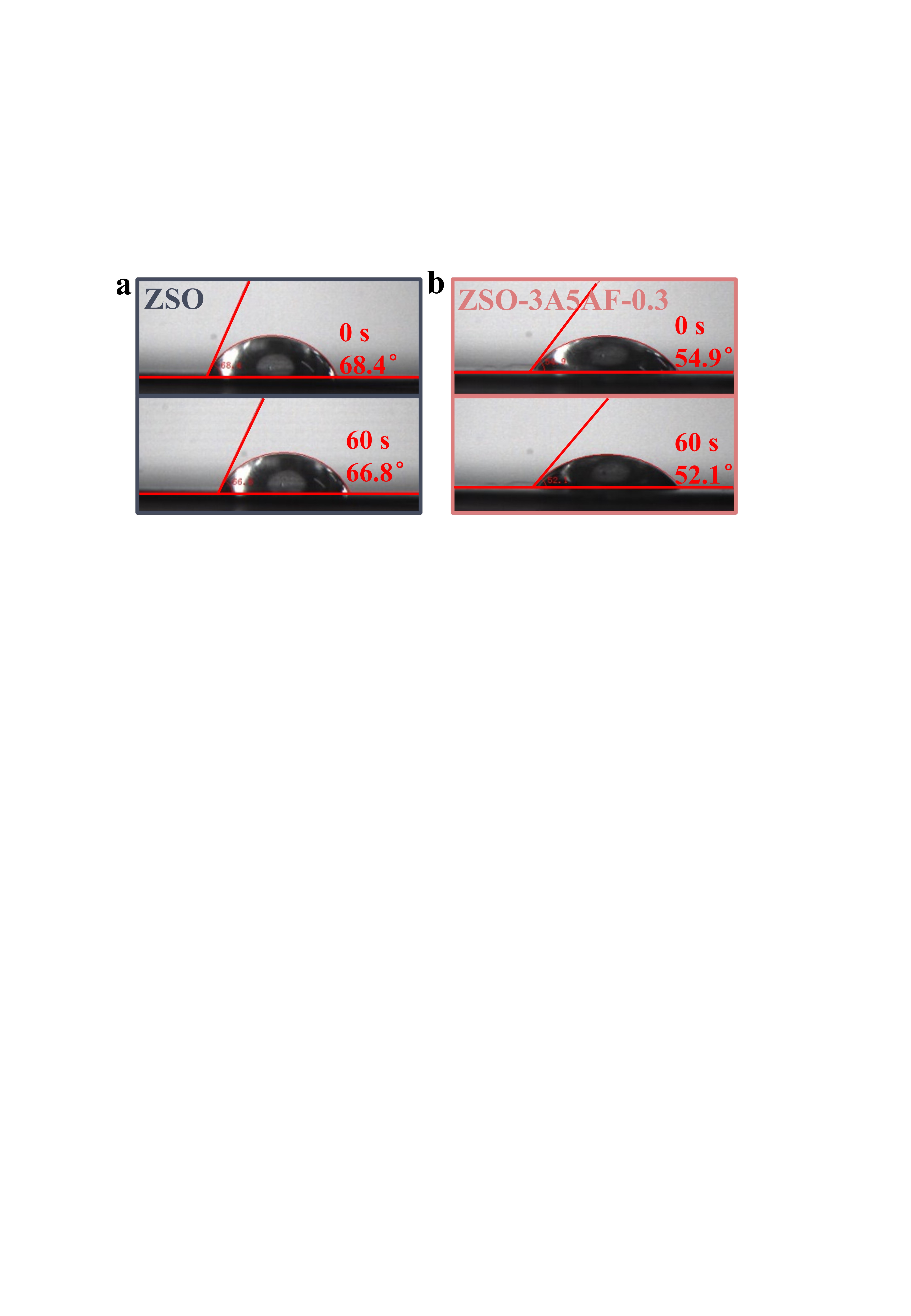


**Figure S6.** Optical images of contact angles of (a) blank ZSO and (b) ZSO-3A5AF-0.3 in initial state and standing for one minute.


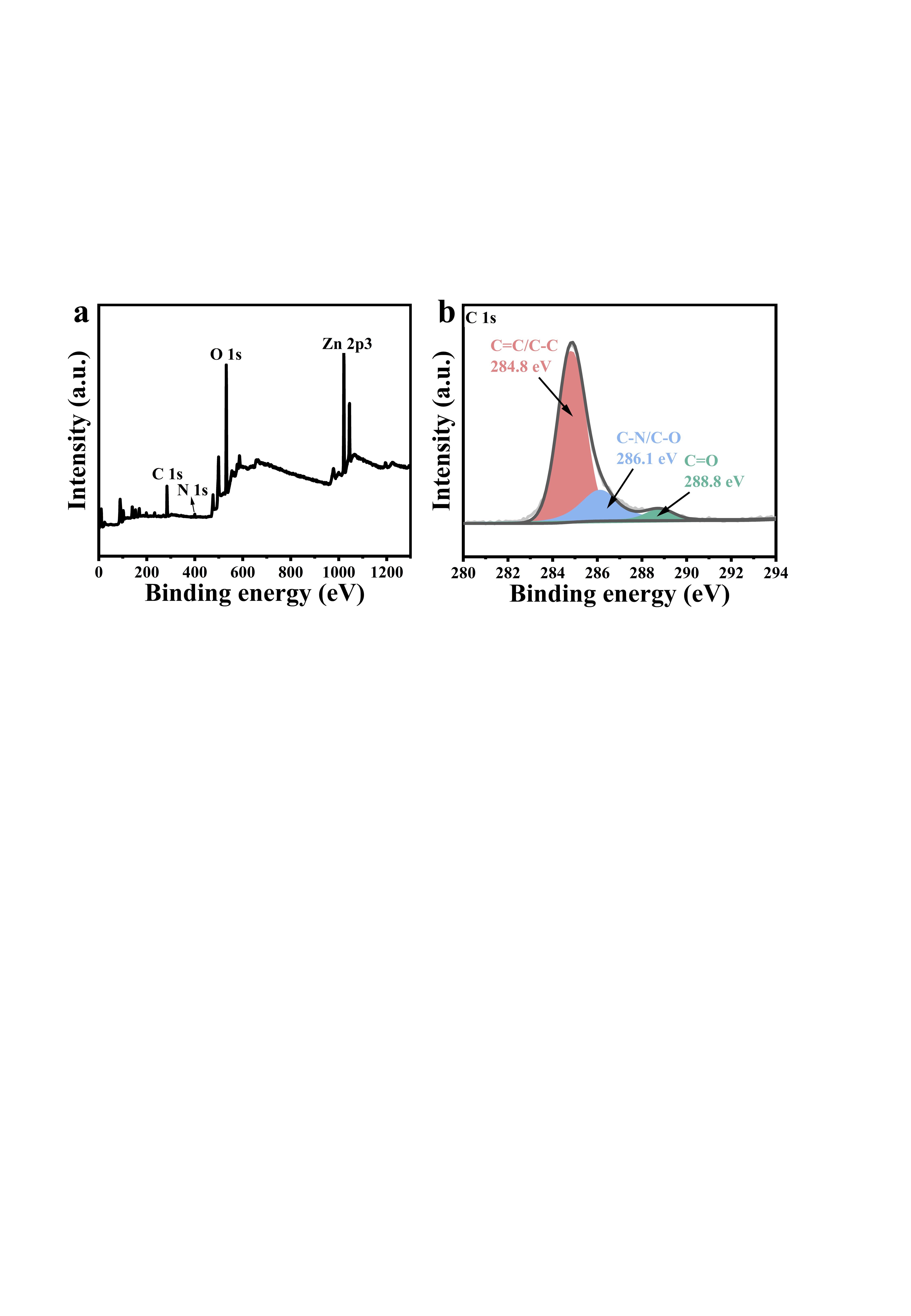


**Figure S7.** The (a) XPS Spectra and (b) C1s high-resolution spectra of zinc foil after 20 cycles in ZSO-3A5AF-0.3 electrolyte.


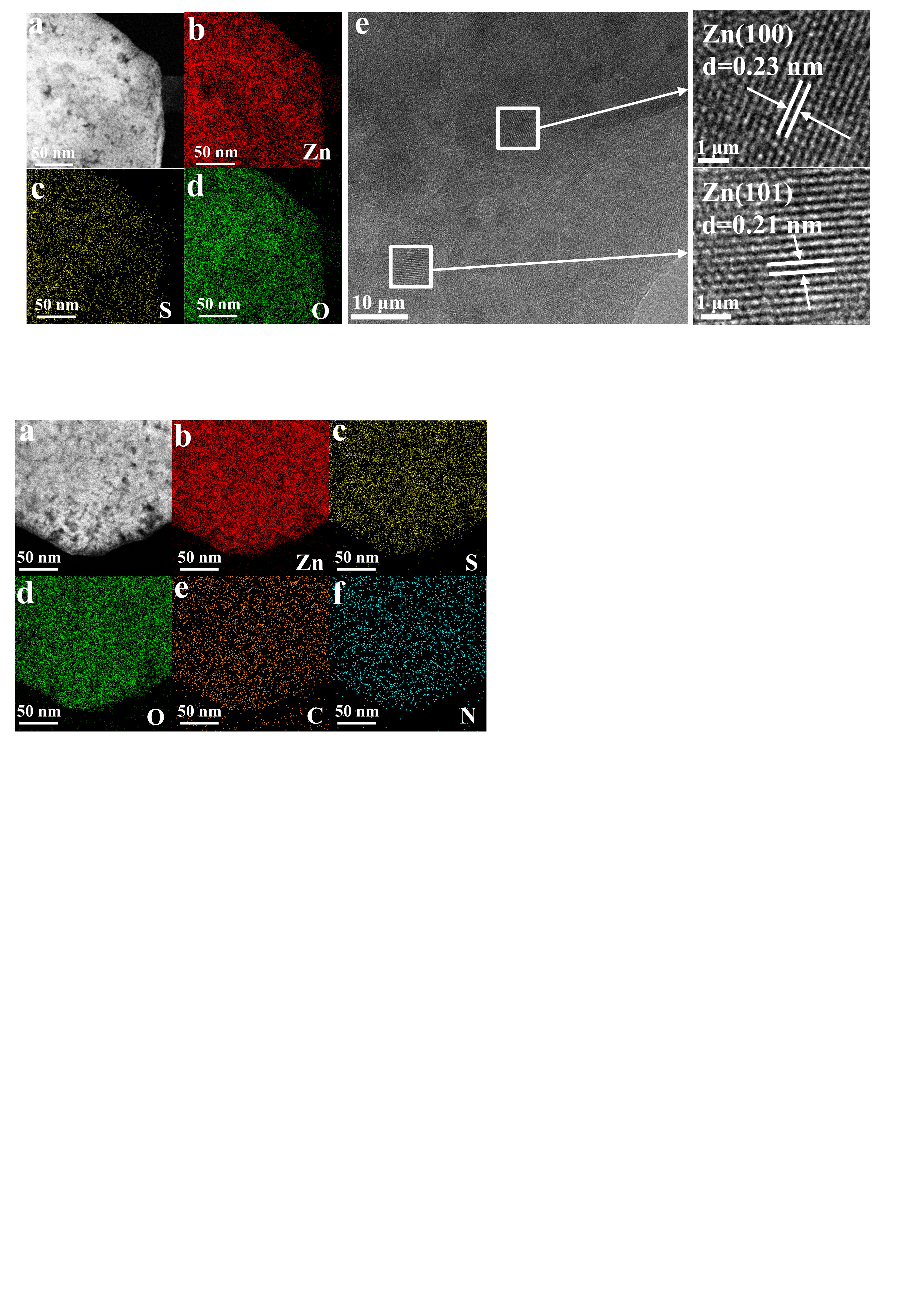


**Figure S8.** (a-f) The corresponding elemental mapping images of the Zn anode cycled in ZSO-3A5AF-0.3 electrolyte.


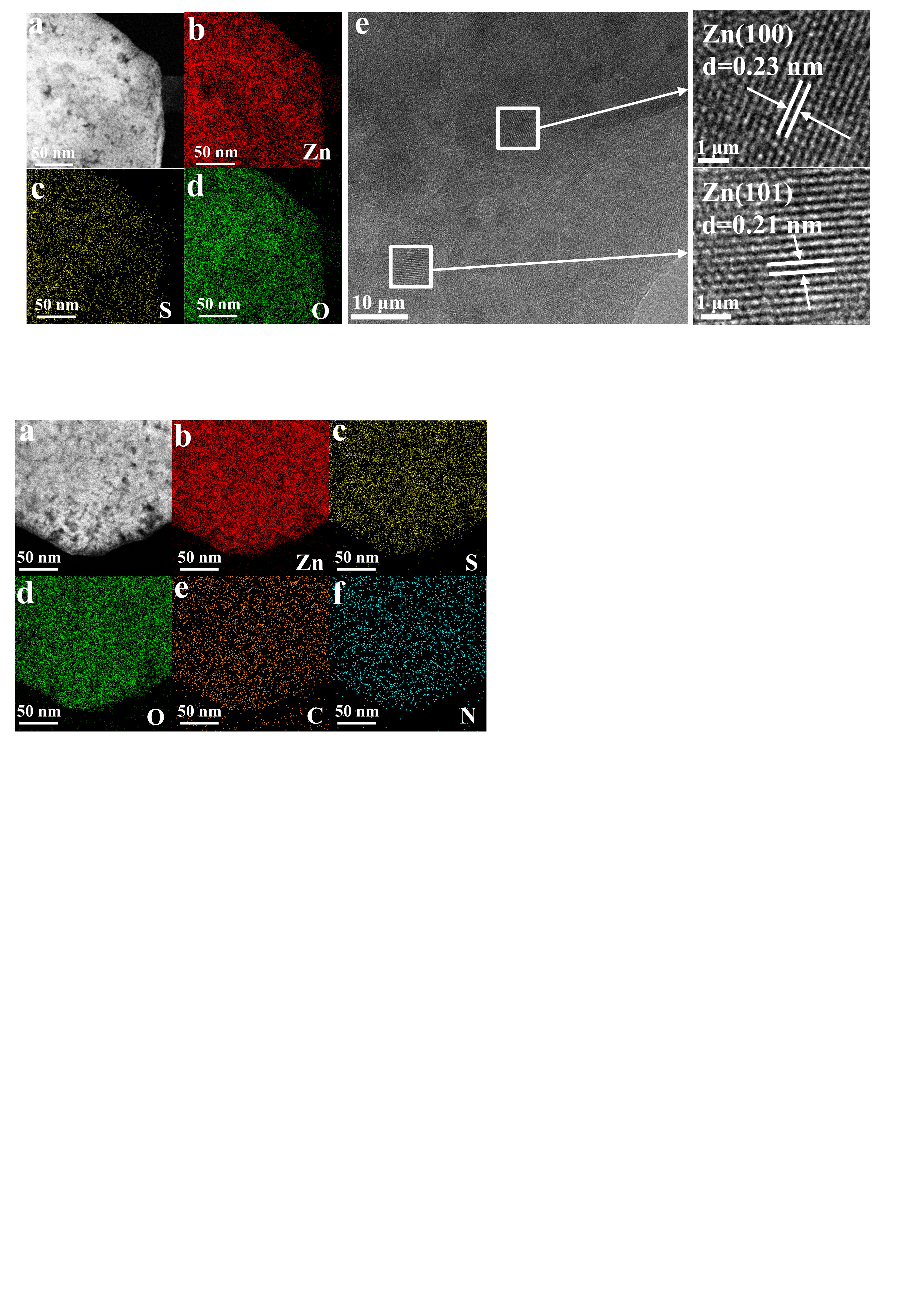


**Figure S9.** (a-d) The corresponding elemental mapping images of the Zn anode cycled in blank ZSO electrolyte. (e) HRTEM image of Zn anode after cycled in blank ZSO electrolyte.


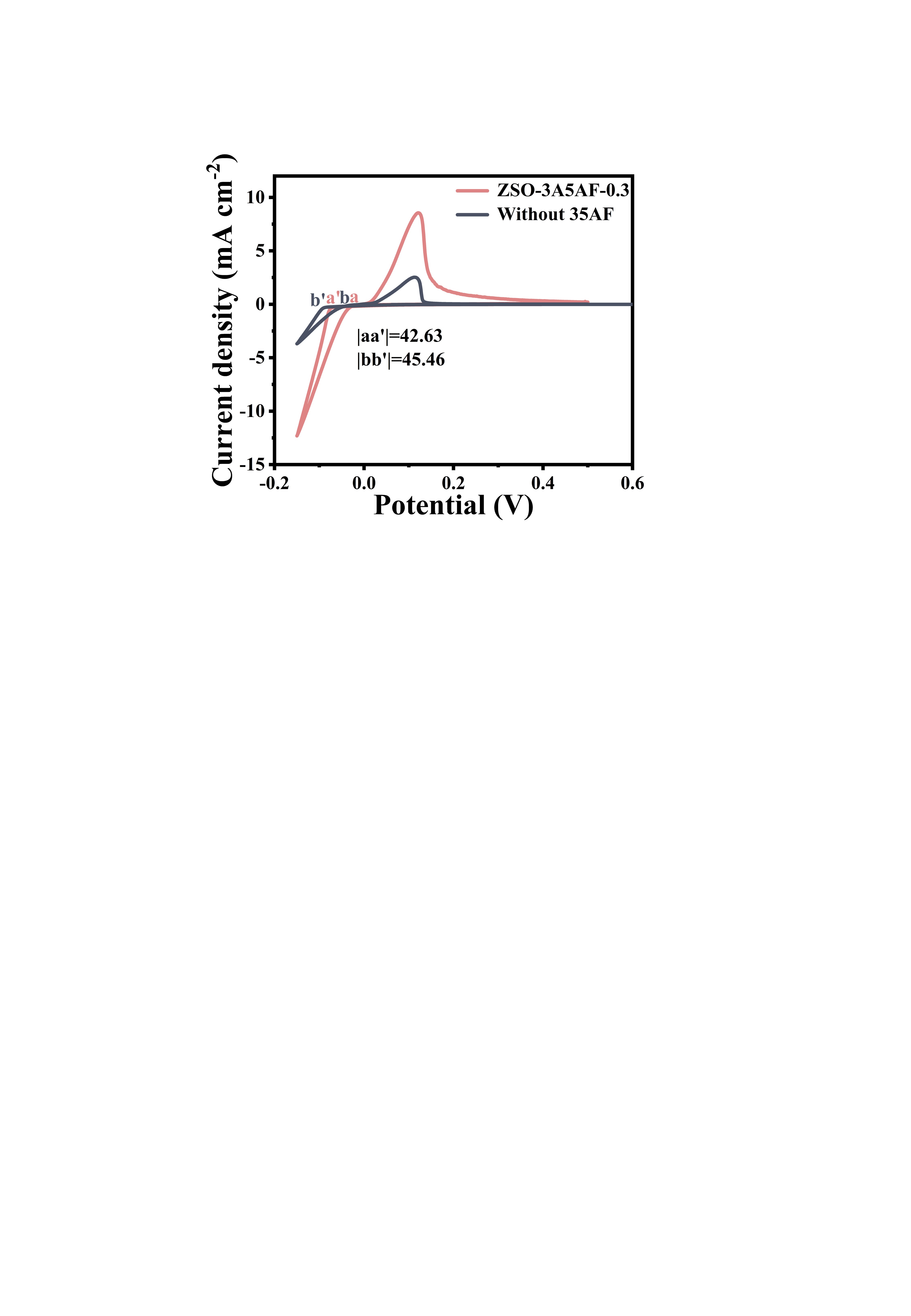


**Figure S10.** CV curves of Zn||Ti half cells were tested in ZSO-3A5AF-0.3 and blank ZSO electrolyte at 1 mV s^-1^ scanning rate.


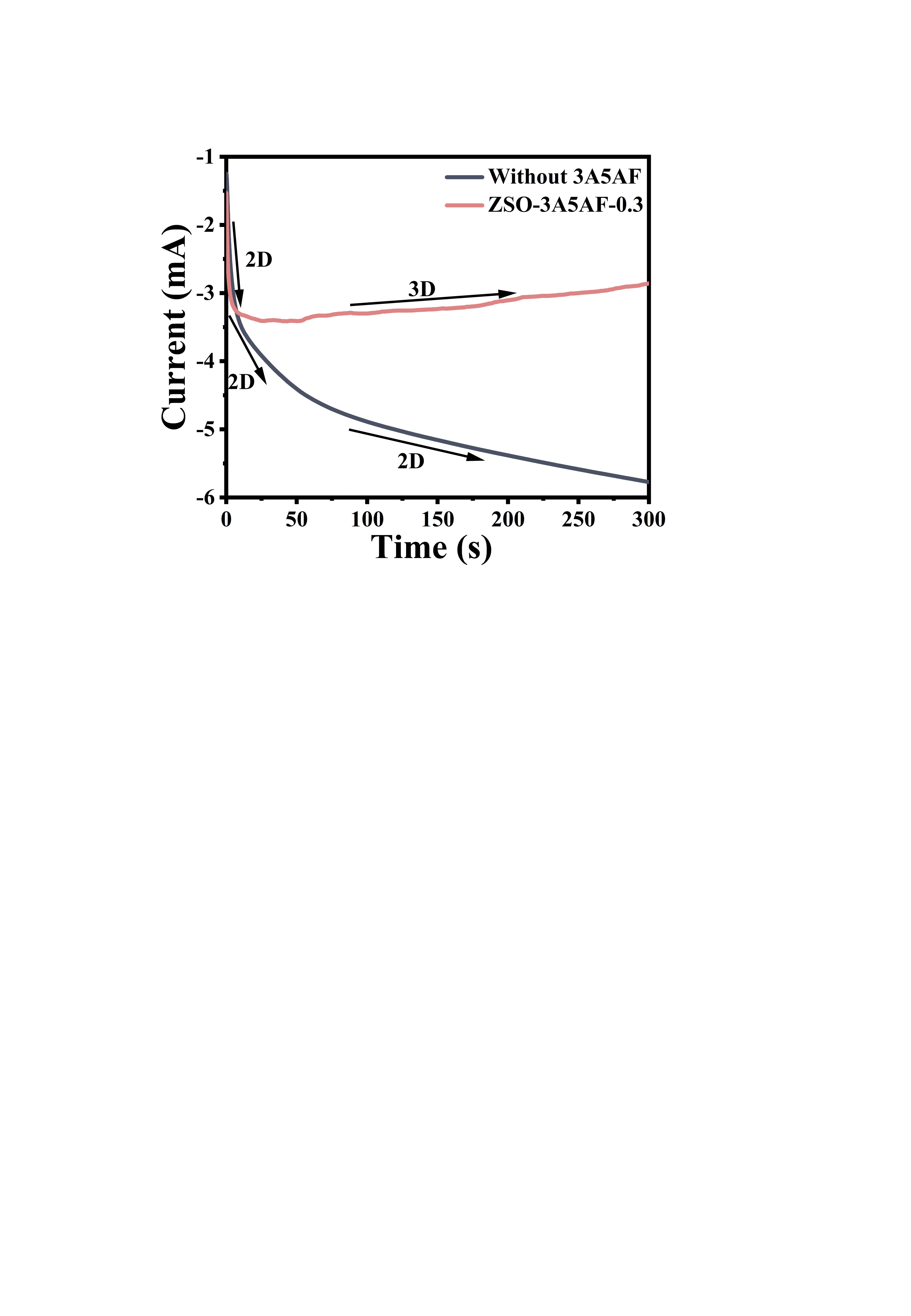


**Figure S11.** CA curves of Zn||Ti half cells in ZSO-3A5AF-0.3 and blank ZSO electrolyte an a fixed voltage of -150 mV.


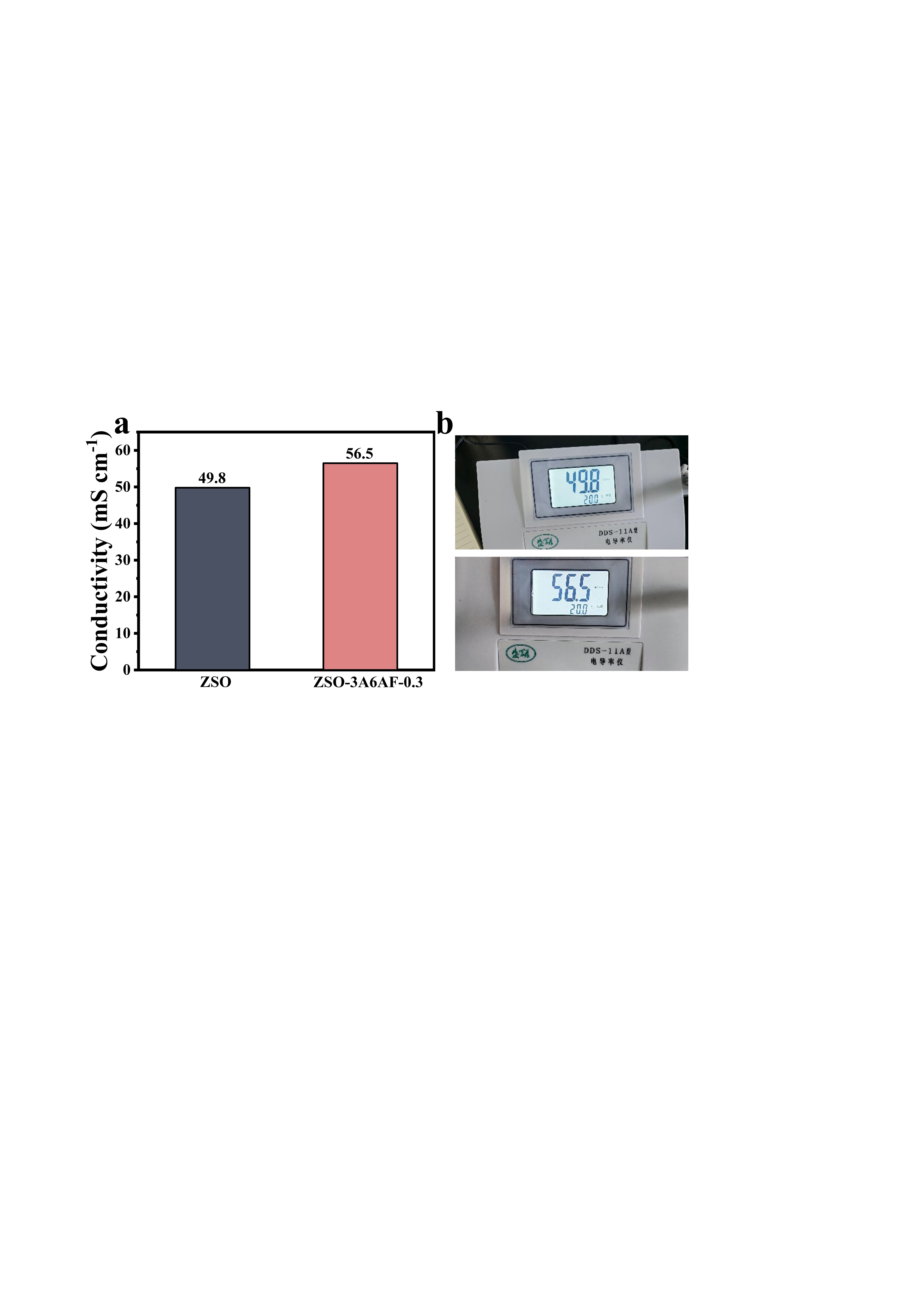


**Figure S12.** (a) Ionic conductivities of blank ZSO and ZSO-3A5AF-0.3 electrolyte and (b) corresponding optical images.


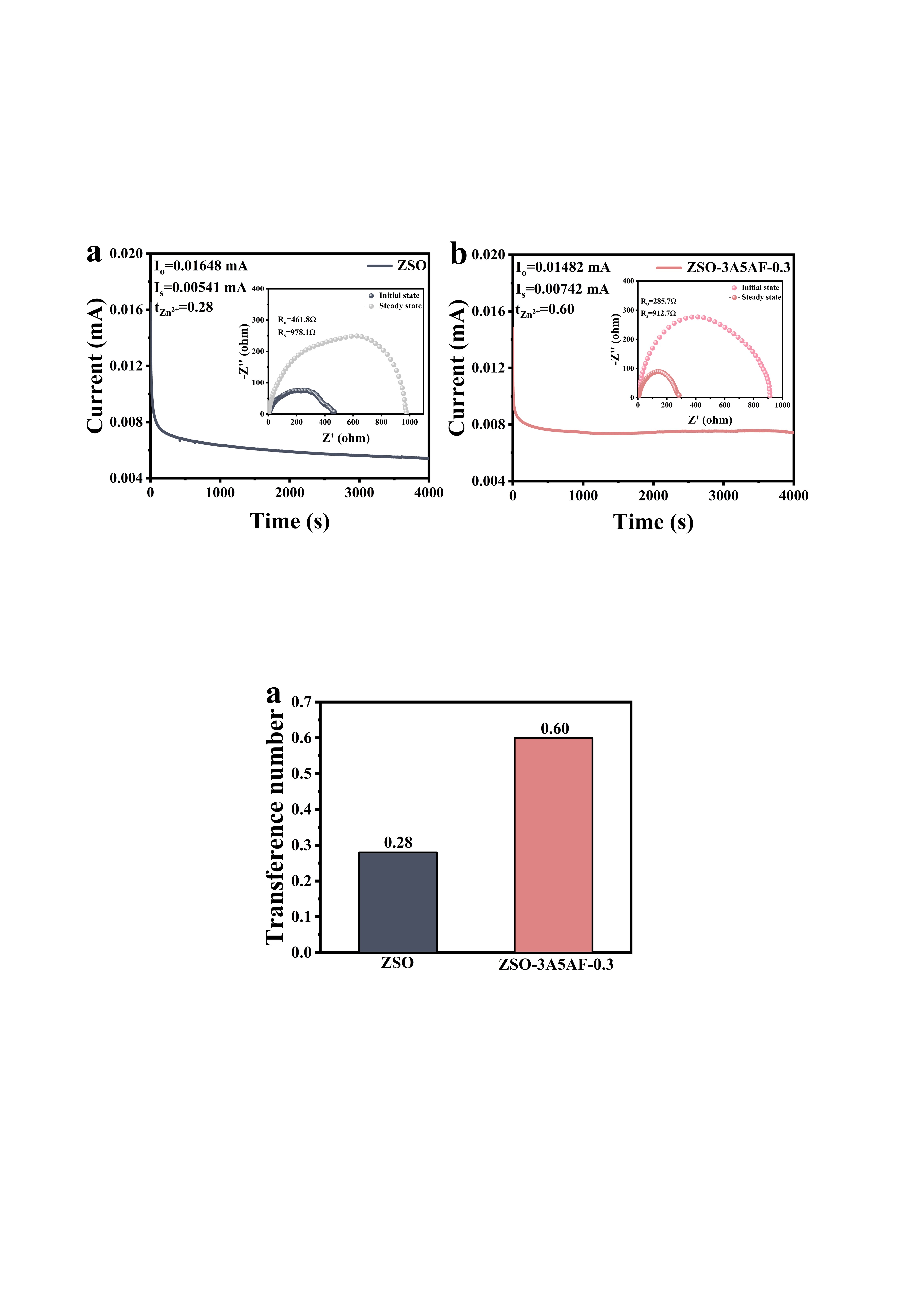


**Figure S13.** Current−time plots of (a) ZnSO_4_ and (b) ZnSO_4_ + 3A5AF symmetric cells after polarization for 4000 s at constant potential (20 mV). The insets are Nyquist plots before and after polarization.


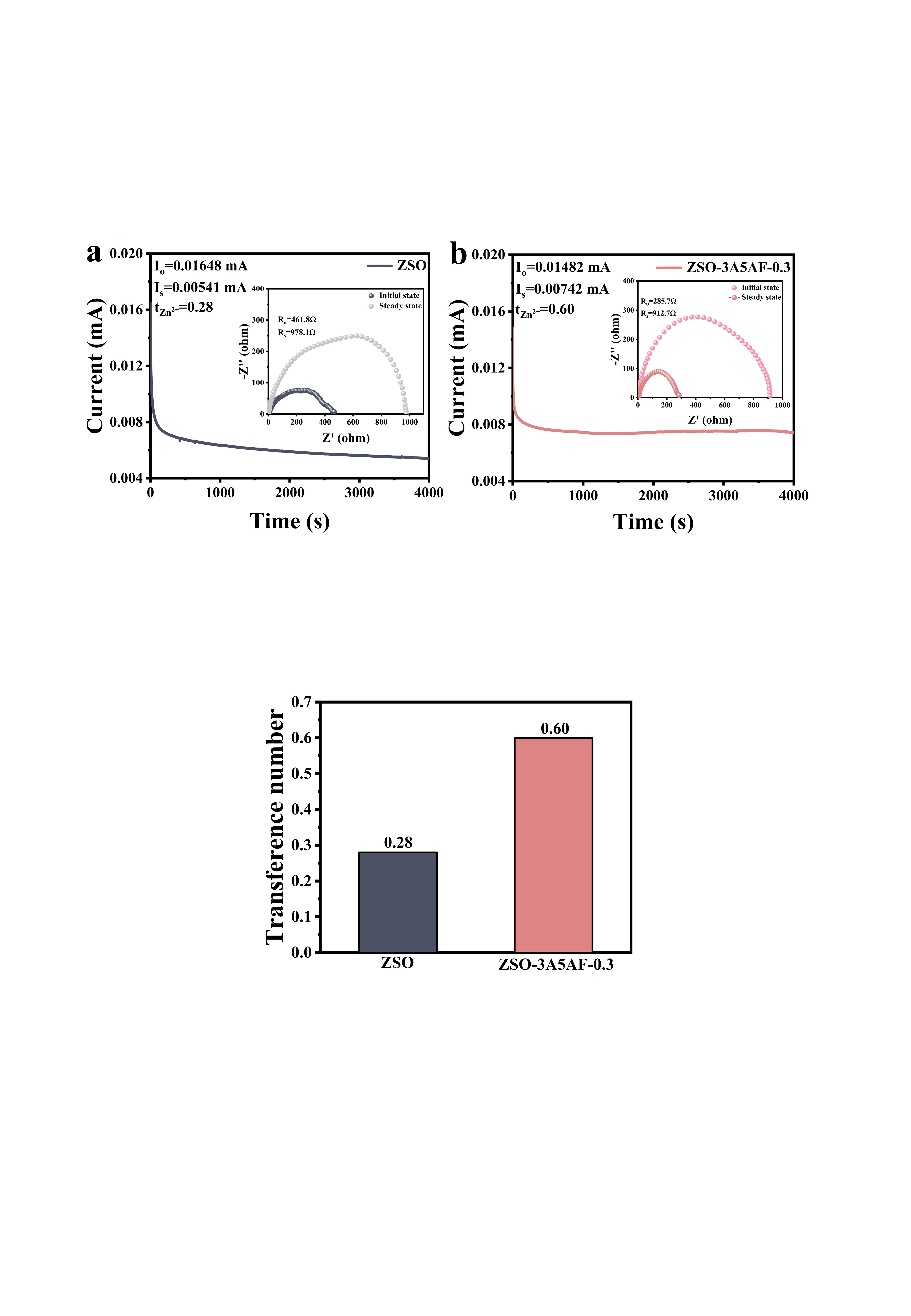


**Figure S14.** Transference numbers of Zn^2+^ in different electrolytes.


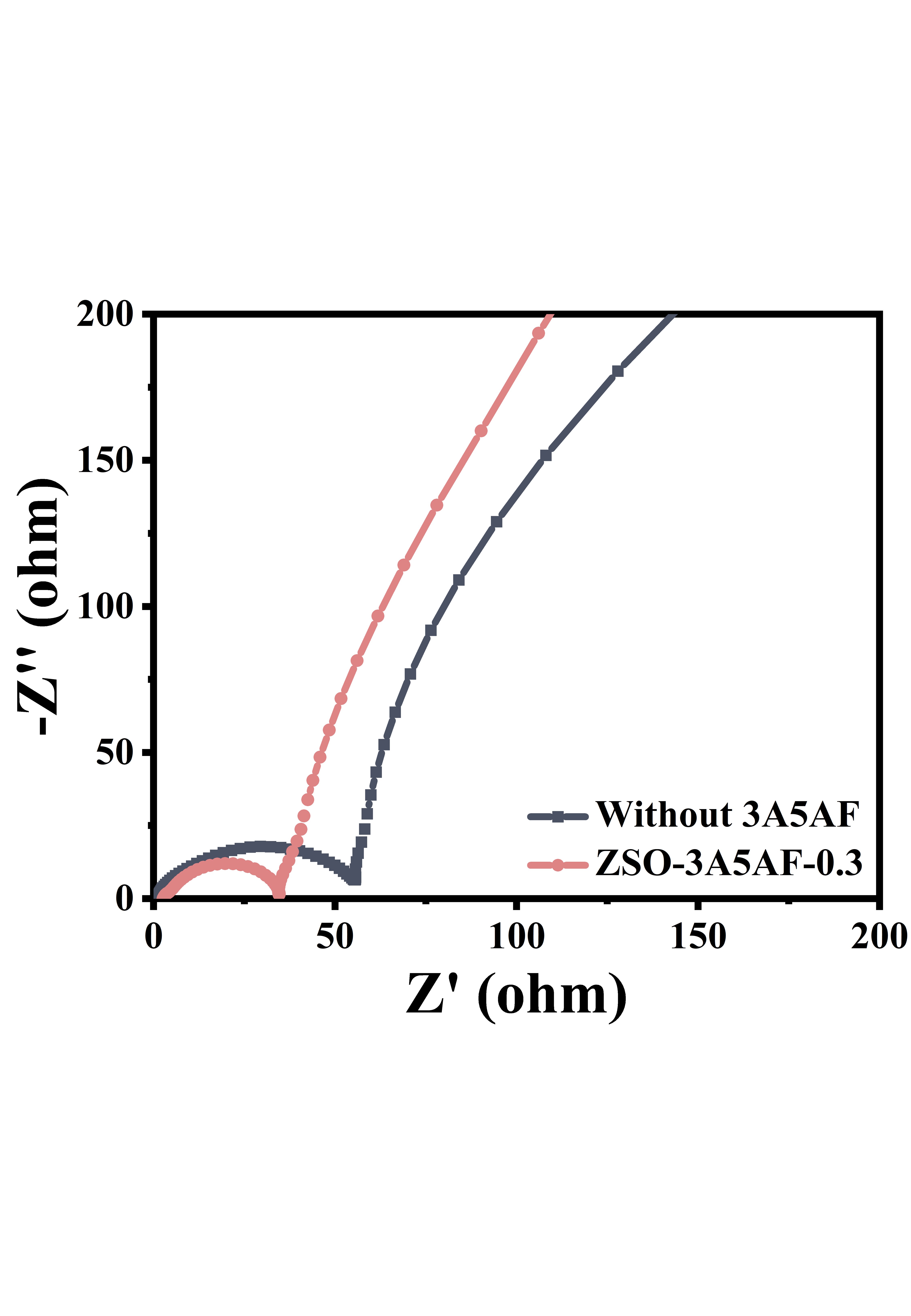


**Figure S15.** EIS spectra of the Zn||I_2_ full cells using the ZSO electrolyte with and without 3A5AF.

**Table S1.** The electrochemical performances of Zn||Zn symmetrical cells with various Additives at low current density.

| **Electrolyte additive** | **Current density**  **(mA cm^-2^)** | **Areal capacity**  **(mAh cm^-2^)** | **Times (h)** | **Ref.** |
| --- | --- | --- | --- | --- |
| TMA_2_SO_4_ | 1 | 1 | 500 | [S1] |
| DMSO | 0.5 | 0.5 | 1000 | [S2] |
| ACN | 1 | 1 | 1300 | [S3] |
| Et | 1 | 1 | 1370 | [S4] |
| NE | 1 | 0.5 | 1500 | [S5] |
| HT | 1 | 0.5 | 1800 | [S6] |
| TXA | 1 | 1 | 2000 | [S7] |
| Sorbitol | 1 | 0.5 | 2000 | [S8] |
| **3A5AF** | **1** | **1** | **2700** | **This work** |

**Table S2.** The effect of different electrolyte additives on the electrochemical performance of Zn||I_2_ battery.

| **Electrolyte additive** | **Discharge capacity** | **Discharge current density** | **Cycle number** | **Ref.** |
| --- | --- | --- | --- | --- |
| ZS+CP5 | 158.6 mAh g^-1^ | 10 A g^-1^ | 1000 | [S9] |
| Pyridine-ZnSO_4_ | 151 mAh g^-1^ | 2 A g^-1^ | 10000 | [S10] |
| LAS+SPES | ≈170 mAh g^-1^ | 0.422 A g^-1^ | 20000 | [S11] |
| ZnSO4+BMIS | 182.1 mAh g^-1^ | 2.11 A g^-1^ | 15000 | [S12] |
| PVEMA | 128 mAh g^-1^ | 2 A g^-1^ | 2000 | [S13] |
| PHT/ZnSO_4_ | 147 mAh g^-1^ | 2 A g^-1^ | 1500 | [S14] |
| ZS-EAc | 149.23 mAh g^-1^ | 4 A g^-1^ | 18000 | [S15] |
| L-CN | ≈200 mAh g^-1^ | 5 A g^-1^ | 3500 | [S16] |
| **3A5AF** | **207.2 mAh g^-1^** | **0.5 A g^-1^** | **1000** | **This work** |
|  | **153.1 mAh g^-1^** | **8 A g^-1^** | **20000** |  |

**References:**

1. H. Cao, X. M. Huang, Y. Liu, Q. Hu, Q. J. Zheng, Y. Huo, F. Y. Xie, J. X. Zhao, D. Lin, *J. Colloid Interface Sci*. **2022**, *627*, 367-374.
2. L. S. Cao, D. Li, E. Y. Hu, J. J. Xu, T. Deng, L. Ma, Y. Wang, X. Q. Yang, C. S. Wang, *J.Am.Chem.Soc*. **2020**, *142*, 21404-21409.
3. J. Q. Shi, K. X. Xia, L. J. Liu, C. Liu, Q. Zhang, L. Li, X. Z. Zhou, J. Liang, Z. L. Tao, *Electrochim. Acta*. **2020**, *358*, 136937.
4. K. Su, X. Y. Zhang, X. Q. Zhang, C. S. Wang, Y. X. Pu, Y. Wang, S. H. Wan, J. W. Lang, *Chem. Eng. J*. **2023**, *474*, 145730.
5. H. Wu, H. T. Yin, H. Tian, J. L. Yang, R. P. Liu, *Energy Environ. Mater*. **2025**, *8*, e12839.
6. B. H. Ren, S. L. Hu, A. Chen, X. Y. Zhang, H. Wei, J. J. Jiang, G. M. Chen, C. Y. Zhi, H. F. Li, Z. X. Liu, *Adv. Energy Mater*. **2024**, *14*, 2302970.
7. J. Y. Yin, H. L. Liu, P. Li, X. Feng, M. H. Wang, C. Y. Huang, M. Y. Li, Y. Q. Su, B. Xiao, Y. H. Cheng, *Energy Storage Mater*. **2023**, *59*, 102800.
8. Y. H. Quan, M. Yang, M. F. Chen, W. J. Zhou, X. Han, J. Z. Chen, B. Liu, S. Shi, P. X. Zhang, *Chem. Eng. J*. **2023**, *458*, 141392.
9. X. Y. Wu, W. T. Wang, X. Chen, J. Xie, X. Y. Li, L. F. Li, M. Zhao, C. S. Li, Y. Z. Piao, M. Chen, *Adv. Funct. Mater*. **2025**, *35*, 2419795.
10. Y. Q. Lyu, J. A. Yuwono, P. T. Wang, Y. Y. Wang, F. H. Yang, S. L. Liu, S. L. Zhang, B. F. Wang, K. Davey, J. F. Mao, *Angew. Chem*. **2023**, *135*, e202303011.
11. R. Wang, Y. Y. Liu, Q. Q. Luo, P. Xiong, X. D. Xie, K. Zhou, W. J. Zhang, L. Zhang, H. J. Fan, C. F. Zhang, *Adv. Mater*. **2025**, *37*, 2419502.
12. H. Wu, J. N. Hao, S. J. Zhang, Y. L. Jiang, Y. L. Zhu, J. H. Liu, K. Davey, S. Z. Qiao, *J.Am.Chem.Soc*. **2024**, *146*, 16601-16608.
13. C. Wu, Y. F. Pan, Y. C. Jiao, P. Y. Wu, *Angew. Chem*. **2025**, *137*, e202423326.
14. Y. C. Guo, H. Wang, H. W. Li, H. T. Wu, P. Xu, J. Xiao, H. W. Wu, *Electrochim. Acta*. **2025**, *523*, 145962.
15. T. Xiao, J. L. Yang, B. Zhang, J. W. Wu, J. L. Li, W. J. Mai, H. J. Fan, *Angew. Chem*. **2024**, *136*, e202318470.
16. H. M. Yu, D. P. Chen, X. Y. Ni, P. Qing, C. S. Yan, W. F. Wei, J. M. Ma, X. B. Ji, Y. J. Chen, L. B. Chen, *Energy Environ. Sci*. **2023**, *16*, 2684-2695.
